# Highly Sensitive and Selective Zinc-Based Metal–Organic Framework Derivatives Gas Sensors for Trace H_2_S Detection

**DOI:** 10.1021/acssensors.5c01743

**Published:** 2025-10-02

**Authors:** Wei Wang, Li Chen, Leif Riemenschneider, Chen-Chen Wang, Luis-Antonio Panes-Ruiz, Martin Hantusch, Yun-Xu Chen, Jian-Jun Zhang, Shivam Singh, Yana Vaynzof, Markus Löffler, Arezoo Dianat, Naisa Chandrasekhar, Shi-Rong Huang, Gianaurelio Cuniberti

**Affiliations:** † Institute for Materials Science and Max Bergmann Center for Biomaterials, 9169 TUD Dresden University of Technology 01062 Dresden, Germany; ‡ University of Strasbourg, Institute Charles Sadron, CNRS, UPR22, 23 Rue du Loess, 67034 Strasbourg Cedex 2, France; § Faculty of Chemistry and Food Chemistry, Technische Universität Dresden, 01062 Dresden, Germany; ∥ Leibniz-Institute for Solid State and Materials Research (IFW), 01062 Dresden, Germany; ⊥ 28286 Max Planck Institute for Microstructure Physics 06120 Halle (Saale), Germany; # Center for Advancing Electronics Dresden (cfaed) and Faculty of Chemistry and Food Chemistry, 28394 TUD Dresden University of Technology 01062 Dresden, Germany; ¶ Chair for Emerging Electronic Technologies, TUD Dresden University of Technology, 01187 Dresden, Germany; ∇ Dresden Center for Nanoanalysis (DCN), Center for Advancing Electronics Dresden (cfaed), TUD Dresden University of Technology, 01069 Dresden, Germany; ○ Dresden Center for Computational Materials Science (DCMS), TUD Dresden University of Technology, 01062 Dresden, Germany

**Keywords:** Zn-MOF, pyridinic nitrogen (PD-N), pyrrolic nitrogen (PR-N), n-doped graphitic carbon, H_2_S gas sensing, DFT

## Abstract

High sensitivity and selectivity are never-ending points of interest in the gas sensing field. Herein, the novel functionalized N-doped graphitic carbon is derived from Zn-MOF by modulating the pyrolysis temperature toward H_2_S sensing application. The results demonstrate excellent sensing performance toward H_2_S gas with a limit of detection (LOD) of 56.9 ppb, faster response and recovery time (18 and 29 s), and high selectivity with a 20-fold response difference than other interfering gases. The expected stability with stable multiple consecutive responses and a strong response toward 1 ppm of H_2_S after 4 months were reached. Functionalized groups pyridinic nitrogen (PD-N) and pyrrolic nitrogen (PR-N) that make MOF-derived carbon stand out in H_2_S gas sensing are mainly attributed to dual active sites: (i) N–C bonds on graphitic carbon undergo surface redox reactions, forming oxidized carbon species (C=O or C=S), and (ii) PD/PR-N-Zn coordination centers facilitate the formation of SO_4_^2–^-based surface complexes through reaction with H_2_S and adsorbed oxygen. Notably, DFT calculation was employed to confirm both PR-N and PD-N bonding with zinc, yielding the largest charge transfer and binding energy among simulated factors, which attributes to the generation of significant sensing performance for H_2_S. Consequently, this work will provide a novel strategy for the advancement of gas sensing applications.

## Graphical abstract

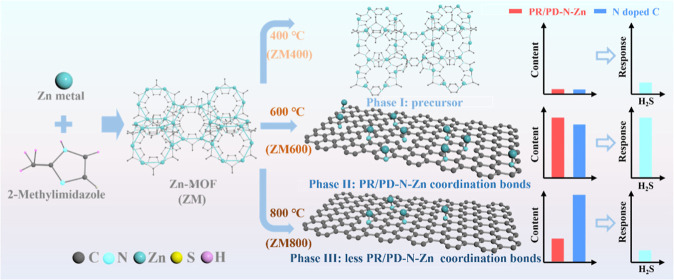

Different levels of hydrogen sulfide (H_2_S) diffused in the environment cause different levels of damage to human health. When exceeding 5 ppm (ppm), H_2_S poses a significant risk to the respiratory system.^1^ As for humans, exposure to 100–150 ppm can result in a loss of sense of smell and 200–300 ppm may lead to pulmonary edema. At levels of 500–700 ppm, individuals may experience loss of consciousness, and concentrations exceeding 700 ppm can result in death within minutes.^2,3^ Therefore, sensors with high sensitivity and selectivity are paramount for addressing such complex challenges effectively.

Metal–organic frameworks (MOFs) have emerged as a functional material for gas sensors due to their large specific surface area, abundance of active sites, customized porosity, controllable morphology, and microstructure.^4–12^ The ZIF (zeolitic imidazolate framework) series is particularly favored for its straightforward synthesis process, structural stability, porous nature, morphological controllability, and cost-effectiveness. In addition to the two most classical MOFs, ZIF67 and ZIF8, which have similar topologies and ligands and exhibit 3D polyhedral structures, another Zn-based MOF named ZIF-L was first reported by Wang et al.^13^ This MOF exhibits a multilayer structure in the microstructure and has a certain size of cavities. While these MOFs exhibit a large specific surface area and numerous active sites, their application in gas sensing is hindered by poor electrical conductivity. To overcome this limitation, MOFs undergo further multifunctionalization via post-treatment to yield MOF-based derivatives. These derivatives inherit the structural benefits of MOFs while simultaneously enhancing active sites through the introduction of heterogeneous atoms, defects, and novel chemical bonds. Additionally, the electrical conductivities of these derivatives are improved. These enhancements render MOF-based derivatives highly valuable for gas sensing applications.^14–32^

The post-treatment of functionalized MOFs can be roughly classified into two strategies: with and without oxygen treatment. With oxygen, MOF-based derivatives usually result in the conversion of metal to metal oxides.^14–26^ Ren et al.^14^ obtained porous ZnO nanoparticles derived from ZIF-8 MOF for NO_2_ gas sensors, which exhibited 51.41% response toward 1 ppm of NO_2_. The results further indicated that mesoporous structures with high channels, specific surface area, and oxygen vacancies are major contributors to sensing performance. Rich oxygen vacancies as the main sensing species were also found in the work of Zhu et al.,^21^ where Al^3+^-Co_3_O_4_ nanocomposites were synthesized derived from Co MOF for n-butanol sensing, presenting a 116.7% response to 20 ppm n-butanol, which is approximately a 5.5-fold improvement compared to pristine Co_3_O_4_. Wang et al.^16^ prepared ZnO/Co_3_O_4_ nanocages derived from Zn/Co-bimetallic MOFs for H_2_S gas sensing. The results presented a 2.0% response toward 500 ppb H_2_S gas at 120 °C and 4% response to 1 ppm of H_2_S. Additionally, the faster response and recovery time (10 and 21 s) were detected, where abundant oxygen vacancies and high specific surface area exert a profound influence. For metal oxides derived from MOFs, in addition to oxygen vacancy species and specific surface area, the main mechanisms in gas sensing include small particle size and porous structure,^15^ spinel structure and metal element composition,^18^ chemical and electronic sensitization of heteroatoms doping,^19^ and heterogeneous structures.^24,25^

Another type of functionalized MOFs is obtained via using protective gases, such as nitrogen or argon, in which metal atoms are encapsulated in carbon-based composites.^27–32^ Fe-doped N-rich porous carbon derived from Zn-MOF was prepared by Zhang et al. toward H_2_S sensing with a limit of detection of 0.13 ppm.^30^ The absorptive surface and voids from the porous carbon skeleton are the primary contributors to the gas sensing performance. The CoZn-NCNTs particles synthesized by Liang et al.^31^ exhibited a 28.9% response toward 30 ppm of SO_2_ at room temperature (RT). The results showed that increased conductivity led by an induced narrow acceptor level with doped Zn boosts sensing performance. Notably, Song et al. investigated how to remove H_2_S from biogas and found that the derived N-doped carbons have strong adsorption properties for H_2_S.^33^ This is a great benefit in the field of sensing. It was investigated by Han et al. that they obtained a Zn–N_*x*_ coordination bond anchored to N-doped carbon materials toward aerobic oxidative cleavage and esterification of C­(CO)–C bonds by pyrolysis of zinc chloride/chitosan composites.^34^ The results indicated that the coordination bond between Zn^2+^ and N changes the electronic state of the metal and the synergistic effect between Zn and its surrounding N together contributes to the catalytic performance. However, the research on the functionalized PR/PD-N in MOF-derived carbon materials focusing on gas sensing has not yet been revealed.

Here, we combine experimental techniques and density functional theory (DFT) simulations to investigate the pivotal function of PR/PD-N-Zn coordination bonds in gas sensing mechanisms and introduce a novel strategy for enhancing the sensor efficacy. In this context, we employ a straightforward annealing technique, leveraging the spindle-shaped Zn-MOF to configure PR/PD-N-Zn coordination bonds immobilized onto nitrogen-doped graphitic carbon to detect H_2_S gas, as depicted in Figure 1. By controlling the temperature to modulate the PR/PD-N-Zn coordination bond, it was found that ZM annealed at 600 °C (ZM600) exhibited excellent H_2_S gas sensing performance in both air and N_2_ conditions, in particular, responding to 1 ppm of H_2_S in air in only 18 s, and achieved a LOD of 56.9 ppb, high selectivity, and stable cycling characteristics, which are the best among MOF-derived carbon materials. To verify the long-term stability of the sensor, a 25% response to 1 ppm of H_2_S was demonstrated after 4 months. This work could provide a novel design perspective for the advancement of gas sensing applications.

**Figure 1:**
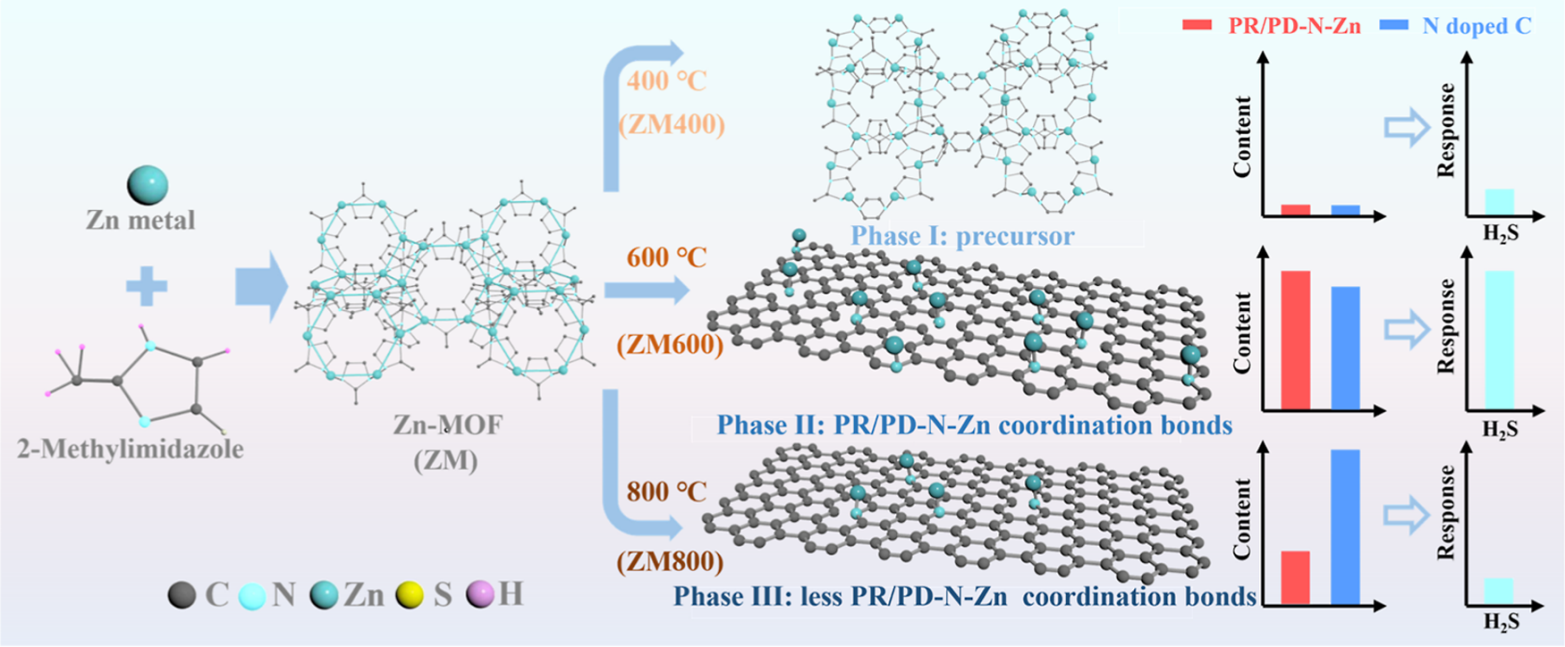
Illustration of the pyrolysis method for preparation of PR/PD-N-Zn functionalized N-doped graphitic carbon toward H_2_S gas sensing.

## Experiments

### Synthesis of Zn-MOF and PR/PD-N-Zn Functionalized N-Doped Graphitic Carbon

The Zn-MOF (denoted as ZM) was synthesized via a simple solution-based method at room temperature. Specifically, 4 mmol of Zn­(NO_3_)_2_·6H_2_O and 32 mmol of 2-methylimidazole were each dissolved in 40 mL of deionized water, respectively. The 2-methylimidazole solution was slowly added dropwise to the Zn^2+^ solution under continuous magnetic stirring at 600 rpm. The mixed solution was stirred at room temperature for 24 h to ensure full coordination reaction. The white precipitate formed was collected by centrifugation (10,000 rpm, 3 min), washed three times with absolute ethanol, and dried in a vacuum oven at 60 °C overnight. All reagents used were of analytical grade and purchased from Thermo Fisher Scientific (Germany), used without further purification.

To prepare PR/PD-N-Zn functionalized nitrogen-doped graphitic carbon materials, the ZM precursor was evenly spread in a quartz boat and placed in a horizontal quartz tube furnace. Prior to pyrolysis, Ar gas (99.999% purity) was flowed through the tube at a rate of 240 sccm for 30 min to remove residual oxygen. The sample was then annealed at target temperatures of 400 °C, 500 °C, 600 °C, 700 °C, and 800 °C for 2 h under a continuous Ar flow. The heating rate was set at 5 °C min^–1^. After being naturally cooled to room temperature, the black powders obtained were denoted as ZM400, ZM500, ZM600, ZM700, and ZM800, respectively.

### Preparation of Sensors and Gas Sensing Measurement

Each of the synthesized samples (2 mg) was dispersed in 2 mL of absolute ethanol and sonicated for 20 min to ensure a homogeneous suspension. After sonication, approximately 10 μL of the middle layer of the suspension was carefully extracted using a micropipette and drop-cast onto an interdigitated electrode (IDE) substrate prepatterned with interdigitated gold electrodes (electrode gap: 3 μm, width: 4 μm) (Figure S1a,b). The samples were dried on a hot plate at 40 °C for 10 min to allow for complete evaporation of ethanol. The fabricated sensor devices (see Figure S1c) were subsequently activated in a vacuum oven at 100 °C for 4 h and stored in a desiccator under dry conditions until use. All syntheses and sensor fabrications were conducted in triplicate to ensure reproducibility.

All sensing measurements were conducted in a sealed 3D printed chamber with a total internal volume of 100 cm^3^. The gas analytes used for sensing, including H_2_S, NO, NH_3_, PH_3_, CO_2_, NO_2_, SO_2_, CH_4_, H_2_, and ethanol (C_2_H_6_O), were commercially obtained from Air Products Company (UK), prediluted in dry nitrogen. Nitrogen and oil-free compressed air were employed as balance gases. No deliberate dehumidification was performed, and the ambient relative humidity (RH) during the tests was maintained at ∼15%. Target concentrations of each analyte were precisely adjusted using a gas mixing and flow control system (MCQ Instruments GB-103) with a total flow rate maintained at 300 sccm. The gas exposure cycle consisted of 100 and 50 s analyte exposure followed by 100 and 50 s recovery in pure carrier gas (nitrogen and air), respectively. The test chamber was sealed and continuously purged with balance gas before exposure to each target gas to ensure a stable baseline.

During testing, the sensor was powered with a constant bias voltage of 1.0 V, and real-time electrical resistance signals were recorded by using a Keithley 2450 source meter. A ceramic microheater (1 cm × 1 cm) was used to control the sensing temperature (room temperature to 250 °C) via a 320-KA3305P DC power supply and real-time temperature monitoring using a Keithley DMM6500 6 1/2-digit multimeter.

Sensor response (S) was defined as the relative resistance change: *S* (%) = Δ*R*/*R*_0_ × 100 = (*R*_0_ – *R*_*t*_)/*R*_0_ × 100, where *R*_0_ is the initial resistance in nitrogen or air and *R*_*t*_ is the resistance upon exposure to the analyte. Response and recovery times were defined as the time to reach 90% of the total resistance change during gas exposure and purging, respectively.

### Characterization

An X-ray diffractometer (XRD) with Cu Kα X-ray radiation was employed to record the crystal structure and purity of as-prepared materials. The morphology and microstructure of materials were measured by a scanning electron microscope (SEM, Zeiss Gemini 500 SEM) with a low operating voltage (≤1 kV) and high-resolution transmission electron microscopy (HRTEM, Cs-corrected FEI Titan 80–300 microscope) with an operating voltage of 300 kV. X-ray photoelectron spectroscopy (XPS, PHI 5600 spectrometer, Al K-monochromated, 300W) was applied to investigate the elemental composition and state of the surface of samples. Fourier transform infrared (FT-IR) spectroscopy was performed on a Bruker Optics ALPHA-E spectrometer with a universal Zn–Se ATR (attenuated total reflection) accessory in the range 400–4000 cm^–1^. An energy-dispersive X-ray spectrometer (Oxford X-Max^N^ 150 mm^2^ detector) was used to detect elemental mappings of as-prepared samples. The XPLORA PLUS Raman spectrometer with an excitation source of an Ar laser (λ = 532 nm) was implemented to obtain the Raman spectra of specimens. Transmission electron microscopy (TEM, Carl Zeiss Libra 200) with an operating voltage of 200 kV, equipped with high-resolution structural and elemental analysis units, was employed to carry out STEM imaging and EELS to support the investigation of Zn species and local coordination environments.

### Computational Details

Static calculations for configurations were performed to investigate the binding energy amount between the gas molecules and oligomerized ASA molecules by using the Semiempirical Extended Tight-Binding GFN2-xTB program package (version 6.6.0).^35^ The docking submodule—the automated Interaction Site Screening (aISS) program was employed to screen out the most favorable binding configurations,^36^ which implements genetic optimization with the rigid intermolecular force field xTB-IFF, followed by GFN geometry optimizations. Mulliken population analysis was applied for determination of the partial charges,^37^ and the DFT-D4 ^38^ was used for the dispersion correction. The binding energy was obtained by the energy difference between the bonded entity and the two isolated states.

## Results and Discussion

### Characterization of the Sensing Materials

Figure 2 depicts the SEM images of both the ZM and ZM600. The ZM exhibits a spindle-shaped nanosheet structure with a length of ∼10 μm and a thickness of ∼100 nm, showing a uniform morphology and a notably smooth and flat surface (Figure 2a,b). By following annealing treatment, the surface of the spindle becomes rougher, while the smaller spindles agglomerate to form larger, porous structures with dimensions of approximately 70 μm (Figure S2). Particularly at 600 °C, numerous small pores were visibly present on the sample surface (Figure 2c,d), facilitating the adsorption and reaction of gas molecules. However, at 800 °C, the morphology again appeared smooth on the surface (Figure S2e1–e3). These changes are primarily attributed to the reorganization of the microstructure at high temperatures, wherein the ZM materials containing Zn–Zn, Zn–N, C–C, C–N, and C–H chemical bonds^13^ undergo the breaking of old chemical bonds and the formation of new bonds, resulting in the formation of small pores as some of the broken ions detach from the material. Furthermore, elemental distribution characterization was performed by means of energy-dispersive X-ray (EDX) mapping on the ZM600 sample (Figures 2e and S3). The results indicate that Zn, C, and N are uniformly distributed in the sample. The transmission electron microscopy (TEM) images of ZM600 (Figures 2f and S4) reveal that the edges of the sample exhibit derived graphitic carbon structures. However, clear diffraction patterns were not observed in the deeper blue areas, partially attributed to the poor crystallinity of the graphitic carbon material and the diversity of its morphology and also to the thickness of the sample, which prevents penetration of the electron beam. These findings are further confirmed by the characterization methods described below.

**Figure 2:**
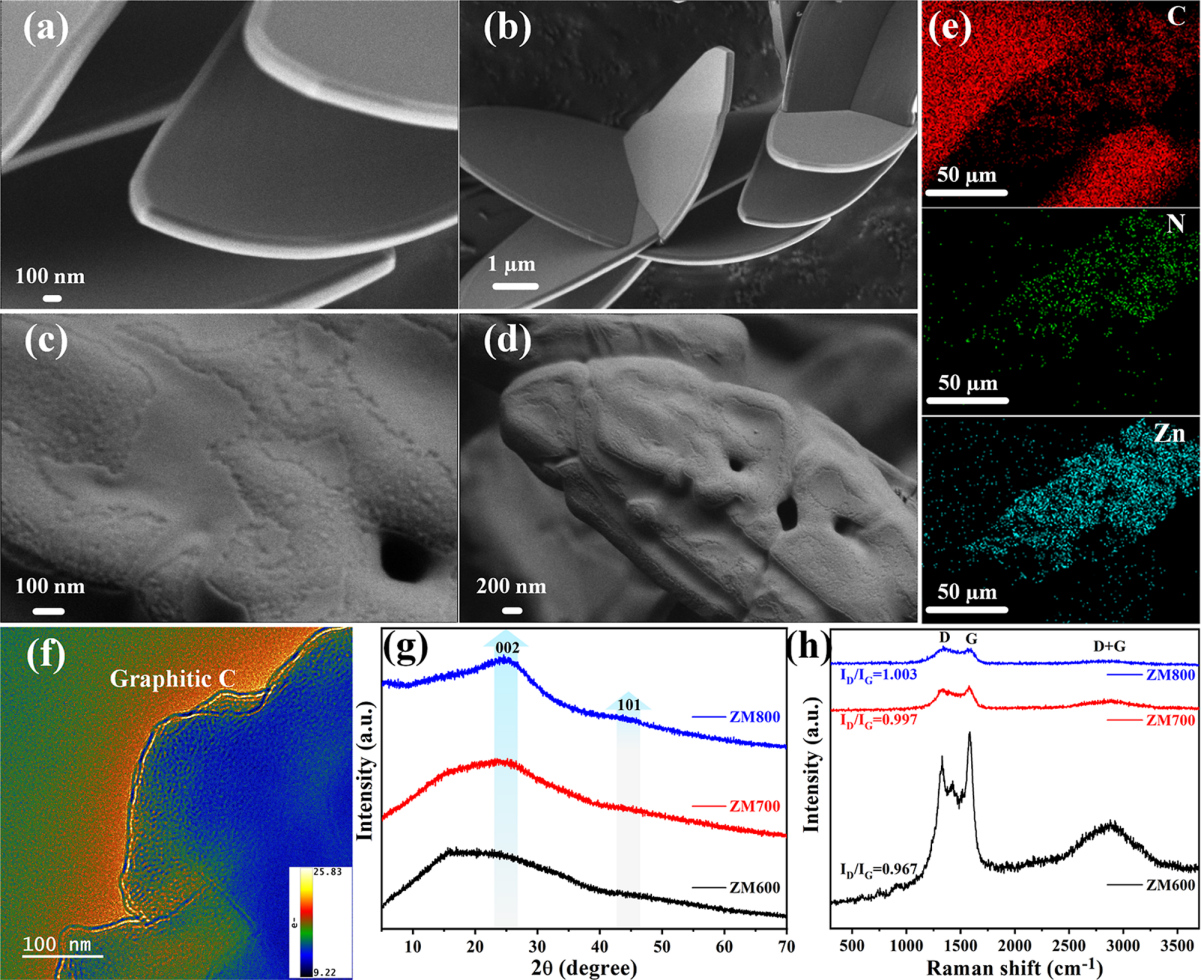
SEM images of (a,b) ZM and (c,d) ZM600, and (e) EDX mapping of ZM600. (f) Brightness depth high-resolution transmittance electron microscopy image of the lattice fringe of ZM600. (g) XRD pattern and (h) Raman spectra of ZM600, ZM700, and ZM800.

Figure 2g displays the XRD patterns of the samples, revealing distinct phase transitions in ZM with increasing annealing temperature. At 400 °C, the XRD results show diffraction peaks different from those of ZM, wherein the breaking of Zn–Zn bonds (1.17 eV) with lower bond energy, part of C–N bonds (2.9–3.4 eV), even C–H (4.2–4.6 eV) bonds initially occurred, then forming new precursor (Figures 1 and S5). Upon annealing at 600 °C, a secondary phase transition takes place, evidenced by the broad peaks at around 2θ = 20° and around 45° corresponding to the (002) and (101) planes of graphitic carbon, respectively. The mechanism involves the methyl group on the five-carbon ring breaking the old bond between the two nitrogen atoms adjacent to it and at the same time recombining with the methyl group outside the ring to form a graphitic carbon (six-carbon ring). As the temperature is further increased to 800 °C, only distinct characteristic peaks of graphitic carbon are observed, indicating the completion of the phase transition. However, no diffraction peaks corresponding to ZnO, Zn metals, or Zn­(CN)_*x*_ are detected in the XRD patterns. These results indicate that upon annealing, N-doped graphitic carbon materials, which exhibit strong adsorption properties toward H_2_S, are formed.^33^ It is known that Zn atoms at different positions may coordinate with different nitrogen atoms (N15, N16, N25, N26, and N35)^13^ to form Zn–N_4_ complexes or Zn–N_*x*_ species, attributed to the existence of zinc in various oxidation states beyond the commonly observed +2 and 0 states, including +3 and mixed valence states.^34–39^

Furthermore, the production of N-doped graphitic carbon was confirmed by Raman characterization. Strong D band and G band characteristic peaks emerge starting from an annealing temperature of 600 °C (Figure 2h), whereas no distinct characteristic peaks were observed at lower temperatures (Figure S6). The characteristic peaks of the Raman spectra of carbon materials, namely, the D band and G band, arise from the vibrational modes of impurities, defects, and amorphous carbon as well as the lattice vibrations of carbon atoms in the material. The presence of the D band typically indicates the presence of amorphous carbon or other structural defects in the material, while the G band represents the presence of crystalline carbon.^40^ Therefore, the intensities of the D and G bands directly reflect the quantity of structural defects and the content of crystalline carbon in the material, respectively. Typically, we employ the ratio of *I*_D_/*I*_G_ to characterize the degree of graphitization of derived carbon materials. It is evident that with increasing annealing temperature, the *I*_D_/*I*_G_ ratio increases, indicating a decrease in graphitization. At 600 °C, the G band is notably higher than the D band, with the minimum *I*_D_/*I*_G_ ratio, illustrating that the ZM600 sample is primarily composed of crystalline carbon.

X-ray photoemission spectroscopy (XPS) was employed to further characterize the composition and chemical states of the as-prepared samples (Figure 3). As shown in Figure 3a, the survey spectra indicate that the predominant elements in the materials are C, N, and Zn. The signal from the onset of O 1s originates from physical adsorption of oxygen on the sample surface. The C 1s spectra (Figure 3b) mainly consist of C–C peaks at approximately 284.8 eV and C–N and C=O peaks with a binding energy of 287.5 and 288.1 eV, respectively.^41,42^ The C=O peak arises from surface contamination during sample loading for the measurement. The spectra indicate that the ZM400 sample (annealed at 400 °C) exhibits a significant presence of C–C with a weak C–N signal. As the annealing temperature increases, the contribution of the C–N species increases. Consequently, nitrogen atoms are incorporated into the derived graphitic carbon in a doped form, as evidenced by the N 1s spectra in Figure 3c, where the signal peak of graphitic nitrogen (GR-N) increases with increasing temperature. Furthermore, the contributions of pyridinic nitrogen (PD-N) and pyrrolic nitrogen (PR-N)^41,43^ diminish at higher temperatures, with the emergence of a notable signal at 399.3 eV, which can be attributed to Zn–N_*x*_ chemical species.^34^ Additionally, the Zn–N_*x*_ peaks undergo a significant shift to lower binding energies, indicating a stronger coordination between the N and Zn atoms. This is further confirmed by examining the Zn 2p spectra (Figure S7), where the Zn doublets shift to higher binding energy with increasing annealing temperature. ZM600 sample exhibits characteristic peaks of two Zn species at 1021.92 and 1044.92 eV. The lower binding energy characteristic peak is typical of ZnO (1022 eV).^44^ However, the presence of ZnO and Zn species was not detected in the HRTEM image (Figure 2f) or XRD pattern (Figure 2g). Therefore, the valence state of the Zn species in the ZM600 sample lies between 0 and +2 and forms coordination bonds with N species.

**Figure 3:**
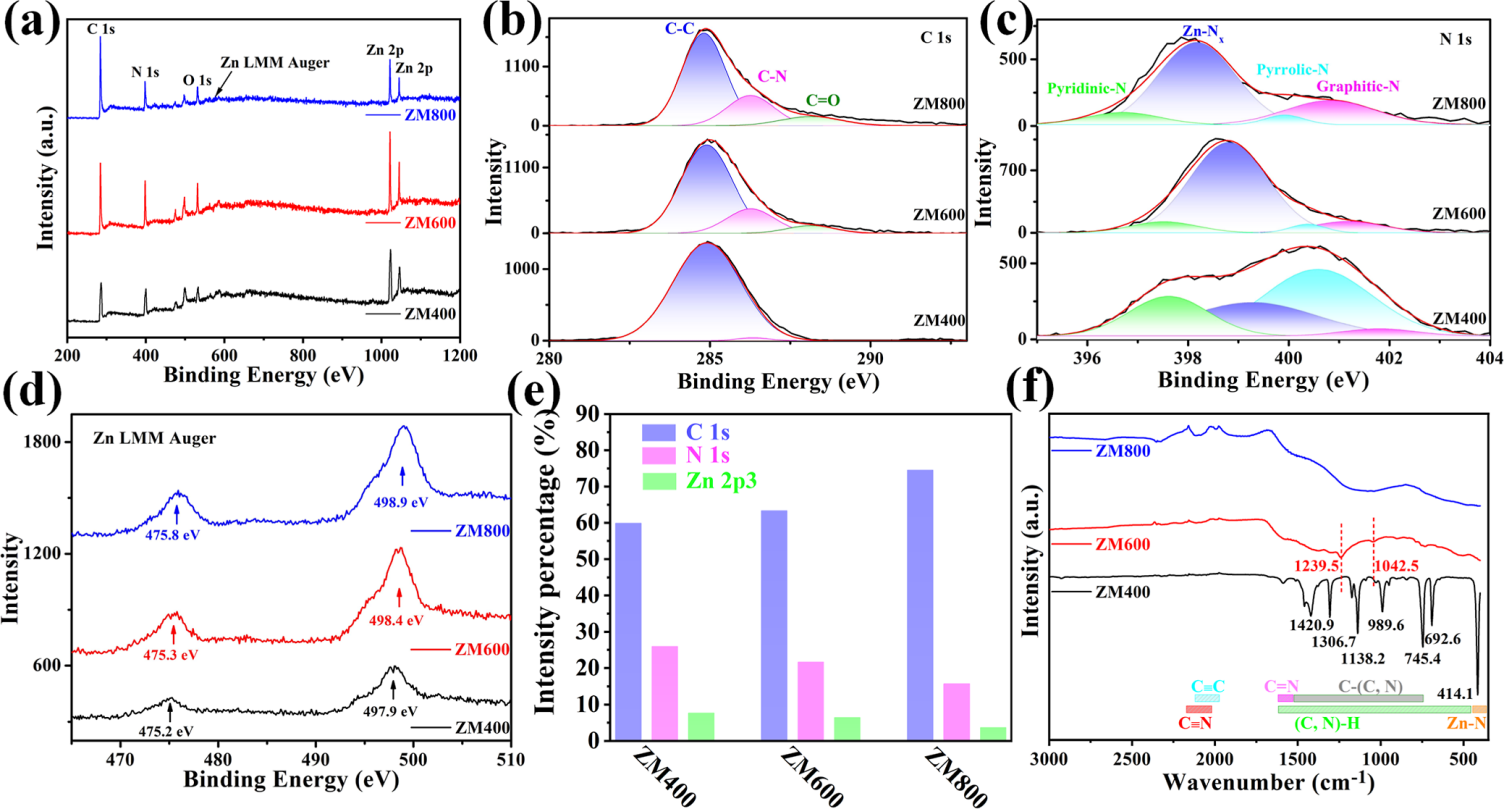
XPS spectra of ZM400, ZM600, and ZM800: (a) XPS survey spectra, (b) C 1s spectra, (c) N 1s spectra, and (d) Zn LMM Auger electron spectroscopy (AES), respectively. (e) Different atomic concentrations and (f) FT-IR spectra of ZM400, ZM600, and ZM800.

Furthermore, Zn LMM Auger Electron Spectroscopy (AES) spectra of the samples were recorded to provide a more intuitive illustration of the changes in the chemical state of zinc after nitridation (Figure 3d). It is evident that the Zn LMM AES undergoes a red shift, indicating the formation of more stable coordination bonds with nitrogen. Such a change is not observable in the Zn 2p orbital spectra as its sensitivity to the specific chemical state of zinc surpasses that of the simple spectra of the Zn 2p orbitals. Additionally, the simple initial spectra shift of the Zn 2p orbitals cannot serve as direct evidence of changes in the chemical state of zinc.^45–47^ All of these results directly alter the electronic states of both nitrogen and zinc, thereby indirectly affecting carbon, which plays a positive role in gas sensing applications.

Moreover, to demonstrate the crucial impact of Zn–N_*x*_ coordination bonds in N-doped graphitic carbon on the gas sensing performance, changes in the relative elemental content in the samples were quantified via XPS. As shown in Figure 3e, the relative contents of carbon, nitrogen, and zinc in the samples are depicted. It can be observed that with increasing annealing temperature, the carbon content in the samples increases, while the content of zinc and nitrogen decreases. At an annealing temperature of 600 °C, nitrogen-doped graphitic carbon is obtained, while the Zn–N_*x*_ species on its surface begin to exert their effects. There is a decrease in the content of zinc and nitrogen, although an increase in N-doped graphitic carbon was occurred in ZM800, which provides direct evidence for exploring the mechanism of sensing performance.

Fourier transform infrared (FT-IR) spectroscopy was also conducted on the as-prepared specimens to elucidate the chemical structure. As depicted in Figure 3f, the ZM400 sample exhibits numerous sharp absorption peaks across different spectral regions. Notably, the absorption peak at 414.1 cm^–1^ corresponds to Zn–N bonds. Typically, metal–ligand bonds in FT-IR spectra appear in the low-wavenumber region (far-infrared region).^48^ Absorption peaks between 692.6 and 989.6 cm^–1^ mainly arise from C–H and N–H bonds, while peaks between 989.6 cm^–1^ and 1500 cm^–1^ correspond to C–C and C–N bonds. Unsaturated C=N bonds are located between 1500 cm^–1^and 1650 cm^–1^, and absorption peaks between 2000 cm^–1^ and 2200 cm^–1^ are attributed to C≡N and C≡C bonds.^49^ Results indicate that with increasing annealing temperature, sharp vibrational peaks including Zn–N bonds disappear. Simultaneously, a prominent broad absorption peak appears in the low-wavenumber region, possibly due to the breaking of existing Zn–N bonds in ZM, resulting in the formation of novel Zn–N_*x*_ coordination bonds with energies exceeding the bond strength. Furthermore, numerous C–H and N–H bonds break, with H^+^ completely dissociating, leading to the reconfiguration of unsaturated C and N bonds as well as Zn and N bonds. Meanwhile, the absorption peaks of unsaturated C≡N and C≡C bonds become increasingly prominent, consistent with the increase in amorphous carbon or other structural defects as demonstrated in the Raman results.

### Gas Sensing Properties of PR/PD-N-Zn Functionalized N-Doped Graphitic Carbon

As shown in Figure 4a, the response and recovery curves of different samples to varying concentrations of H_2_S under N_2_ conditions are depicted. The results indicate significant variations in gas sensing performance among samples annealed at different temperatures. With increasing annealing temperature, the response of the samples to H_2_S gas shows an initial increase, followed by a decrease. The sample annealed at 600 °C exhibits the highest response performance, with a response of nearly 70% to 100 ppm concentration of H_2_S among all as-prepared samples (Figure S8a). This could be attributed to the porous nature of N-doped graphitic carbon with generated Zn–N_*x*_ coordination bonds, confirmed by the TEM image (Figure 2f) and N 1s XPS spectra (Figure 3c), coupled with a higher degree of graphitization (Figure 2h). Combining the analyses from Figures 4a and 3e, it can be distinctly observed that the reduction of zinc and nitrogen contents directly leads to the deterioration of sensor performance, even though the amount of the N-doped graphitic carbon matrix increases. These results indicate that Zn–N_*x*_ coordination sites play a crucial role in the sensing mechanism by facilitating effective charge transfer upon gas adsorption. Although N-doped graphitic carbon can physically adsorb a large amount of H_2_S gas,^33^ this adsorption alone does not significantly contribute to the resistance change and thus has a limited impact on the sensing response.

**Figure 4:**
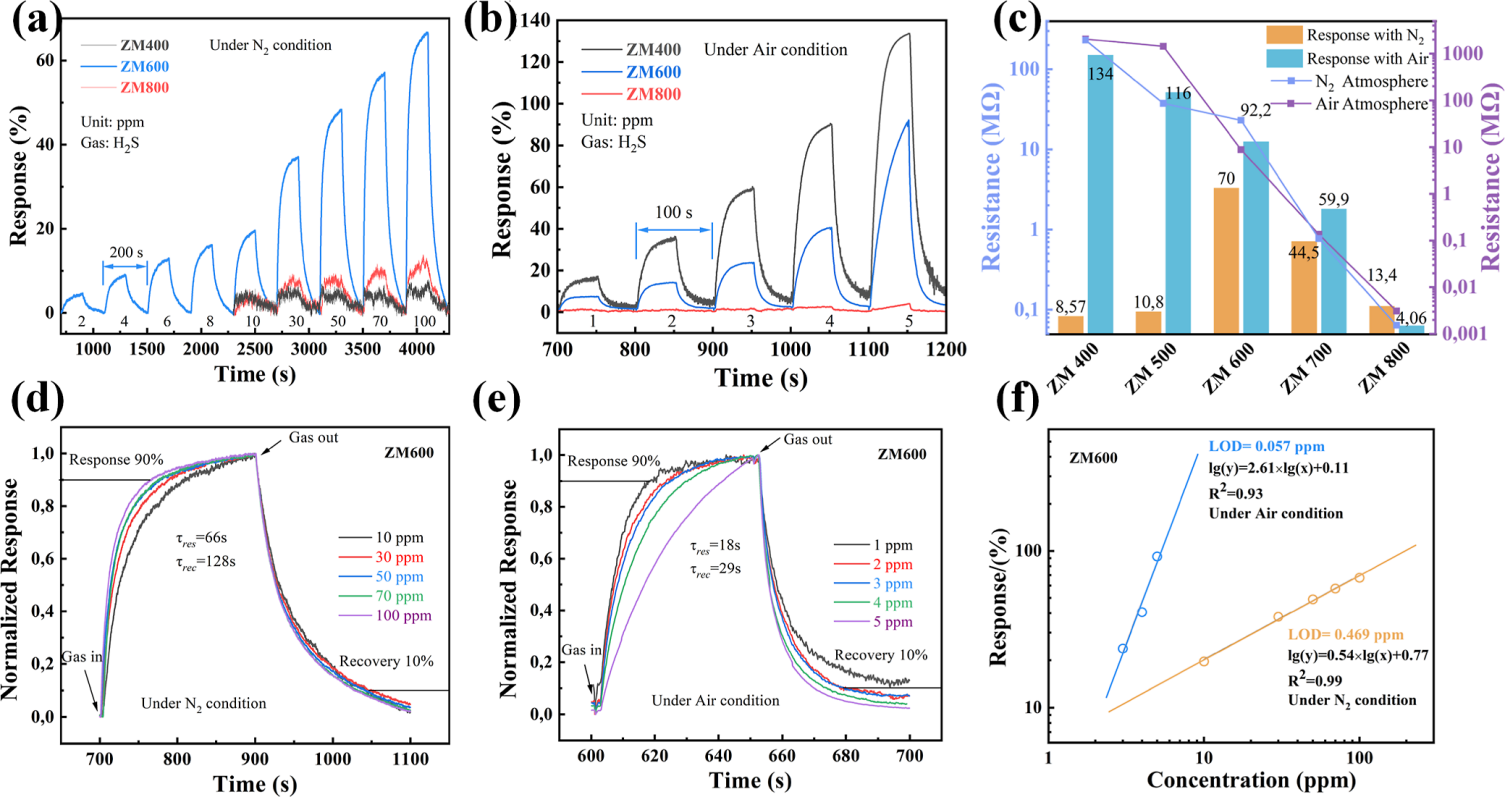
(a,b) Sensing performance of sensors at 200 °C operating temperature: response and recovery curves toward H_2_S with different concentrations under N_2_ conditions (a) and air conditions (b) for ZM400, ZM600, and ZM800. (c) The relationship of response value and resistance of ZM400, ZM500, ZM600, ZM700, and ZM800 to 100 and 5 ppm concentrations of H_2_S under N_2_ and air conditions, respectively. (d,e) Response and recovery time toward a 100 ppm concentration of H_2_S under N_2_ conditions (d) and 5 ppm under air conditions (e). (f) Response concentration log–log plots to different concentrations of H_2_S under N_2_ and air conditions.

This response was obtained with N_2_ as the background gas, effectively eliminating any potential influences from air, particularly oxygen. This approach allows for a precise focus on the sensing mechanism of the porous nature of N-doped graphitic carbon with generated Zn–N_*x*_ coordination bonds. Subsequently, the sensor was also measured under air conditions, as shown in Figures 4b and S8b. At lower H_2_S concentrations (1–5 ppm), both ZM400 and ZM600 exhibit enhanced performance compared to those in N_2_ conditions. Notably, under air conditions, the sensor achieves nearly complete response and recovery within 100 s, which is half the time observed in the N_2_ condition. These results confirm that N-doped graphitic carbon with generated Zn–N_*x*_ coordination bonds plays a key role in the sensing mechanism.

Under N_2_ conditions, the limited sensing performance of ZM400 is attributed to the fact that only the N-doped graphitic carbon with Zn–N_*x*_ coordination bonds contributes to the sensing response. In contrast, under air conditions, both the N-doped graphitic carbon and the chemisorbed reactive oxygen species are involved, resulting in enhanced performance, which enables ZM400 to demonstrate excellent sensing performance even at low H_2_S concentrations. This critical structural unit is generated in abundance at annealing temperatures above 600 °C, resulting in the optimal sensing performance observed in ZM600. At higher annealing temperatures, the amount of this structural unit decreases, which suppresses the performance, as indicated by the response curve of ZM800 in Figure 4a. However, ZM600 shows a rapid and sensitive response even at low H_2_S concentrations under air conditions (Figure 4b). ZM800 also exhibits noticeable response and recovery at 5 ppm of H_2_S—half the minimum detectable concentration under N_2_—but its performance remains significantly lower than that of ZM600 under similar conditions. These comparative experiments confirm that the sensing mechanism is driven by N-doped graphitic carbon with Zn–N_*x*_ coordination bonds, underscoring the sensor’s robust application versatility.

The relationship of the response value and resistance of as-prepared specimens under both N_2_ and air conditions is shown in Figure 4c. It can be seen that all of the samples show a decreasing trend in resistance under N_2_ and air conditions. When the annealing temperature is below 600 °C, the ZM precursor undergoes a first-phase transition, but the generated phases exhibit high resistance characteristics, despite the apparent observation of a porous framework microstructure.

Under air conditions, the observed high baseline resistance (*R*_0_) of ZM400 and ZM500 corresponds well with their enhanced gas response performance. The sensitivity (*S*) is defined as *S* (%) = (*R*_0_ – *R*_*t*_)/R_0_ × 100 in this work. According to this definition, a larger *R*_0_ value can amplify the relative response signal, even if the absolute resistance change (Δ*R* = *R*_0_ – *R*_*t*_) remains comparable. The high *R*_0_ of ZM400 and ZM500 arises from their increased oxygen adsorption, which generates more active oxygen species on the surface. These species effectively react with H_2_S, leading to a larger Δ*R* as well. As a result, both the numerator (Δ*R*) and the denominator (*R*_0_) in the response formula synergistically contribute to enhanced sensitivity. Additionally, a higher *R*_0_ implies a lower baseline current, making the material more responsive to changes in the resistance upon target gas exposure. Similar correlations between baseline resistance, surface properties, and sensing response have been reported in previous studies,^14^ where optimal sensitivity was achieved at intermediate crystallinity levels balancing active sites and carrier mobility. As a key sensing unit, the oxygen molecules are adsorbed onto the surface of the sensing material and capture electrons from the conduction band, forming chemisorbed oxygen species (O^2–^, O_2_^–^, and O^–^) and creating an electron depletion layer, thereby increasing the material’s baseline resistance. When exposed to H_2_S, a highly reducing gas, these oxygen species react with H_2_S molecules, leading to the release of trapped electrons back into the conduction band and causing a pronounced decrease in resistance. This reaction mechanism is strongly oxygen-dependent, as the presence of surface oxygen species is crucial for facilitating electron transfer and enabling a large resistance change.^50,51^

In contrast, under a N_2_ atmosphere where oxygen adsorption is minimal or absent, the depletion layer formation is significantly suppressed, resulting in a limited response to H_2_S. Thus, the sensor relies primarily on the interaction between the N-doped graphitic carbon with Zn–N_*x*_ coordination bonds and H_2_S to induce resistance changes for sensing. These findings demonstrate that surface-adsorbed oxygen plays a pivotal role in the sensing mechanism by modulating the charge carrier dynamics upon exposure to target gases. As a result, the high resistance leads to weaker current signals, with less noticeable current variation from gas responses, which hampers the sensing performance. Conversely, when the annealing temperature exceeds 600 °C, the degree of graphitization of ZM increases, leading to excessively high conductivity, which in turn masks the poor resistance changes induced by gas responses. Both excessively high and low resistance characteristics directly impact the performance of sensors. Notably, the ZM600 maintains a suitable resistance value, which attribute to the highest response among all samples. The resistance of the sample increases upon the introduction of H_2_S gas and decreases upon its removal (Figure S9).

Furthermore, to elucidate the enhanced sensing performance of ZM600 toward H_2_S gas more clearly, Figure 4d,e illustrates the response–recovery curve of the ZM600 sample toward different concentrations of H_2_S under N_2_ and air conditions, respectively. The results indicate a relatively slow response time of 66 s under N_2_ conditions, defined as the time taken for the sensor to reach 90% of its stable response from the point of gas exposure, and a relatively longer recovery time of 128 s, defined as the time taken for the sensor to recover to 10% of its response after gas removal. In contrast, under air conditions, the results show that the response to 1 ppm of H_2_S concentration occurs within 18 s, with recovery completed in 29 s. The ultrafast response and recovery of the sensor in air can be primarily attributed to the combined effects of oxygen species from the air and the N-doped graphitic carbon with Zn–N_*x*_ coordination bonds structural units. These results confirmed active Zn–N_*x*_ coordination sites in ZM600, as well as nitrogen doping into the graphitic carbon conductive network architecture, which acts as both electron donors and hole donors. When H_2_S molecules come into contact with the abundant active sites on the material surface, rapid charge transfer occurs, leading to the redistribution of electrons within the material and rapid changes in conductivity facilitated by the highly conductive network structure. However, as H_2_S molecules diffuse into the porous material and adsorb and bind with numerous active sites, the detachment of these gases requires a longer time during the sensor recovery phase.

To determine the LOD of the ZM600 sensor, response concentration log–log plots under N_2_ and air conditions, respectively, were conducted for different concentrations of H_2_S (Figure 4f). The results indicate a good linear relationship in the range of 10–100 ppm, with a LOD of 469.2 ppb under N_2_ conditions, which was determined based on a signal-to-noise ratio (S/N) of 3, where the concentration at which the signal reaches three times the background root-mean-square (RMS) noise (Figure S10) is defined as the detection limit.^52^ Under air conditions, the calculated LOD was 56.9 ppb. Additionally, we directly tested the LOD of the ZM600 sensor experimentally. As shown in Figure S11, the response–recovery curve of the ZM600 sensor under low concentrations of H_2_S gas (0.1–1 ppm) was measured at the lowest possible levels within the limits allowed by the equipment. The results demonstrate that the ZM600 sensor exhibits a significant response and recovery capability to 0.3 ppm of H_2_S. These results directly prove the ability of ZM600 as a sensing material to detect and sense harmful gas H_2_S at the parts per billion level in practical applications.

In addition, the response of ZM600 toward 3 ppm concentration of H_2_S at different operating temperatures under N_2_ and air conditions was assessed, as shown in Figure 5a. The samples exhibit an optimal response at 200 °C under both conditions. It is evident that the response under air conditions is superior to that under N_2_. The sensing response increases with temperature and reaches a maximum at 200 °C, beyond which it declines slightly at 250 °C. This trend is attributed to the coupled effects of the gas adsorption and desorption dynamics. At moderate temperatures, Zn–N coordination sites provide effective chemisorption centers for H_2_S, while the N-doped carbon matrix ensures rapid electron transport, leading to enhanced response. However, at higher temperatures, excessive thermal energy accelerates the desorption of H_2_S and reaction intermediates, shortening their residence time on the surface. This temperature-dependent behavior has been similarly observed in Zn-based H_2_S sensors.^53,54^ Meanwhile, thermal agitation may also disrupt the synergistic interaction between the Zn–N sites and the conductive network, collectively resulting in a reduced sensing signal.

**Figure 5:**
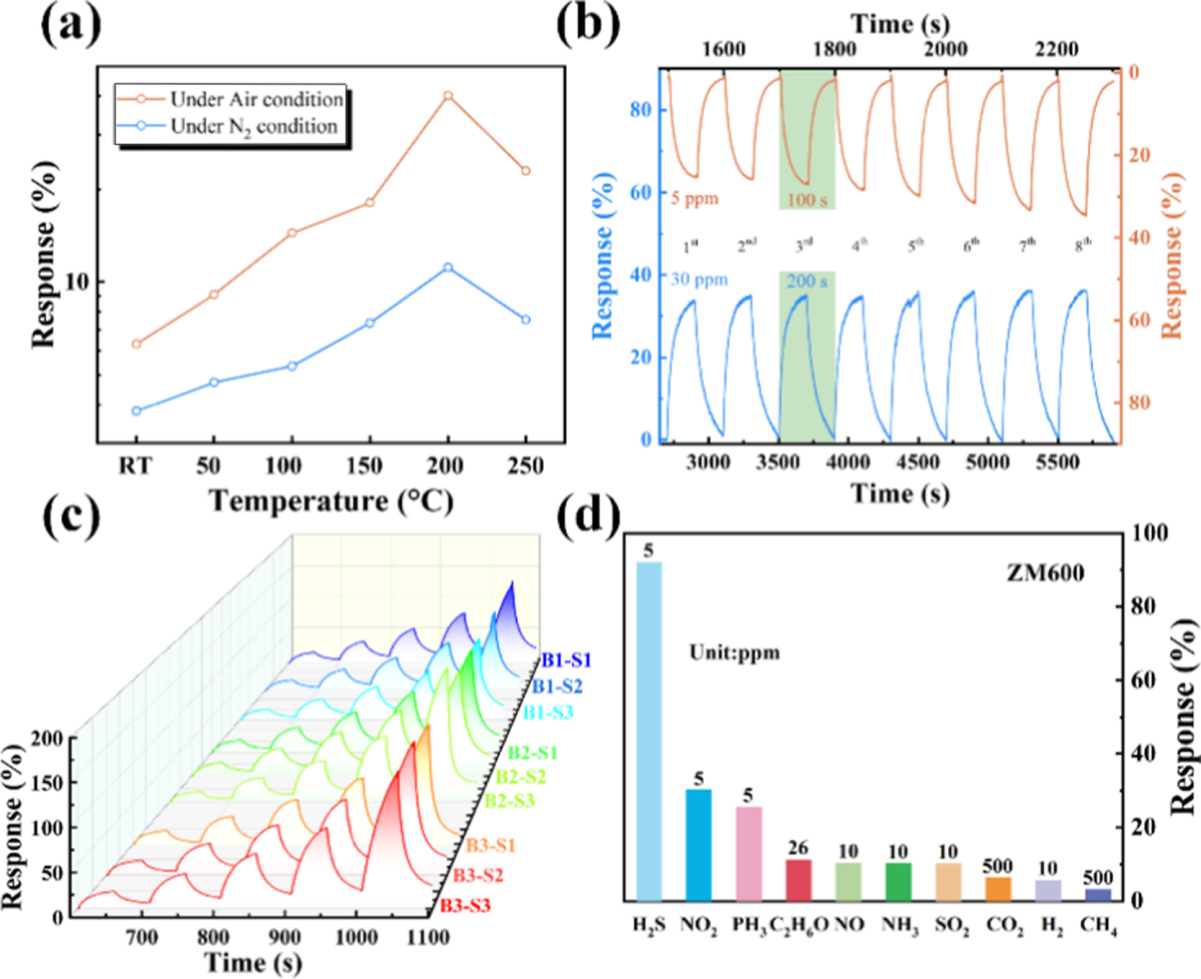
(a) Response of the ZM600 toward 3 ppm at different operating temperatures under N_2_ and air conditions. (b) Recycle sensing toward 30 ppm of H_2_S under N_2_ conditions and 5 ppm of H_2_S under air conditions during eight cycles for ZM600. (c) Response and recovery curves of three sensors from each of three independent batches of ZM600 material, clearly evaluating uncertainties and sensor-to-sensor reproducibility across multiple batches toward H_2_S. (d) A response comparison of ZM600 toward H_2_S and interfering gases at different concentrations.

The stability of the ZM600 sensor in a continuously working condition was confirmed by a cyclic sensing test toward 30 and 5 ppm of H_2_S under N_2_ and air conditions, respectively, over eight cycles (Figure 5b). To rigorously validate the reproducibility and reliability of the sensing performance, three independent batches of ZM600 material were synthesized under identical reaction conditions. For each batch, three separate sensor devices were fabricated by using the same deposition and activation protocol. All devices were evaluated under identical gas exposure conditions. As shown in Figure 5c, the response–recovery curves are consistent both within and across batches. Minimal variation in response magnitude and kinetics is observed, as further confirmed in Figure S12. In addition, SEM, XRD, and Raman spectra of the three batches (Figures S13–S15) reveal nearly identical morphology, crystallinity, and graphitization degree, underscoring the robustness of the synthetic method and sensor assembly process. These results confirm the reliability of the material synthesis process and its suitability for reproducible sensor fabrication, providing a clear evaluation of uncertainties and sensor-to-sensor reproducibility across multiple batches of materials. Long-term stability was demonstrated by the ZM600 sensor, which was stored in a dry cabinet for 120 days and subsequently tested for H_2_S sensing across various concentrations (0.01–10 ppm) (Figure S16). It is found that not only does it exhibit a 25% response to 10 ppm of H_2_S, but it also shows a noticeable LOD as low as 10 ppb of H_2_S.

To assess the gas selectivity of ZM600 toward ten different analytes at representative concentrations were extracted and compared, as shown in Figure 5d. The sensor exhibited the highest sensitivity toward H_2_S (92.16% at 5 ppm), significantly outperforming responses to other gases, even those tested at higher concentrations. For instance, NO_2_ and PH_3_, both tested at 5 ppm, yielded much lower responses of 30.24% and 25.41%, respectively. Meanwhile, gases like C_2_H_6_O, NO, NH_3_, and SO_2_ (tested at 10–26 ppm) all showed comparable responses around 10%, and CO_2_ and CH_4_, even at high concentrations (500 ppm), induced negligible responses of only 6.36% and 3.10%, respectively. These findings demonstrate that the sensor is capable of distinguishing H_2_S from a wide range of potential interferents with high reliability. Moreover, the complete dynamic response–recovery curves at multiple concentrations for all interfering gases are provided in the Supporting Information (Figure S17) to ensure a thorough and transparent data set.

To further correlate the structural characteristics with gas sensing behavior, nitrogen adsorption–desorption analysis was performed, and the results are provided in the Supporting Information (Figure S18). The general trend observed is that the BET surface area increases as the annealing temperature increases. Interestingly, the samples exhibiting intermediate surface areas also demonstrate the best sensing performance. This suggests that while surface area contributes to gas adsorption capacity, it is not the sole determining factor. In particular, the ZM600 sample with optimal sensing performance also exhibits a favorable balance between the surface area and the preservation of Zn–N_*x*_ coordination bonds. These Zn–N_*x*_ coordination bonds play a crucial role in facilitating charge transfer and the reactive activation of H_2_S molecules. Thus, the synergistic effect of accessible porous texture and chemically active Zn–N–C frameworks contributes significantly to the observed sensing enhancement.

Both the presence of chemically active sites and the accessible surface area are essential to ensuring efficient gas–solid interaction during sensing. The chemical composition of derivatives of Zn-MOFs annealed at different temperatures was confirmed using the aforementioned characterization methods (TEM, XRD, Raman, and XPS). Especially for the ZM600 sample, which exhibits the best sensing performance toward H_2_S, its main structure comprises N-doped graphitic carbon with numerous Zn–N_*x*_ coordination bonds. To demonstrate the dominant adsorption sites of H_2_S molecules on the material surface and the effective interaction between them, XPS characterization was performed on the ZM600 sample both before and after H_2_S sensing under both N_2_ and air conditions, shown in Figure 6. After H_2_S exposure, the C 1s spectrum (Figure 6a) exhibits an increase in the C–N peak intensity and the emergence of a new peak around ∼289 eV. This signal may correspond to oxidized carbon species, potentially arising from C=O or C=S functionalities formed during surface redox reactions.^55,56^ Although precise peak attribution around ∼289 eV is challenging due to potential overlap between C=O and C=S species, the increase in this peak intensity—alongside chemical shifts in N_2_ and air conditions—suggests the formation of oxidized carbon species, which may include C=O and/or C=S functionalities formed via surface redox reactions with H_2_S. This is further supported by O 1s spectra, which show increased intensity consistent with oxidized species. In addition, N_2_ was used as the background gas in the experiment, which may contribute to the increase in the C–N bonds. Consistent with the C 1s spectra results, the peak of PR-N increases in the N 1s spectra (Figure 6b), supporting the notion that nitrogen functionalities are actively involved in surface chemistry. Notably, with the newly added air-exposed sample, both C 1s and N 1s spectra exhibit further enhanced peak intensities compared to those of the N_2_ case. This reinforces the conclusion that oxygen facilitates deeper activation and bonding transformation processes on the material surface, thereby amplifying the chemical changes associated with H_2_S exposure.

**Figure 6:**
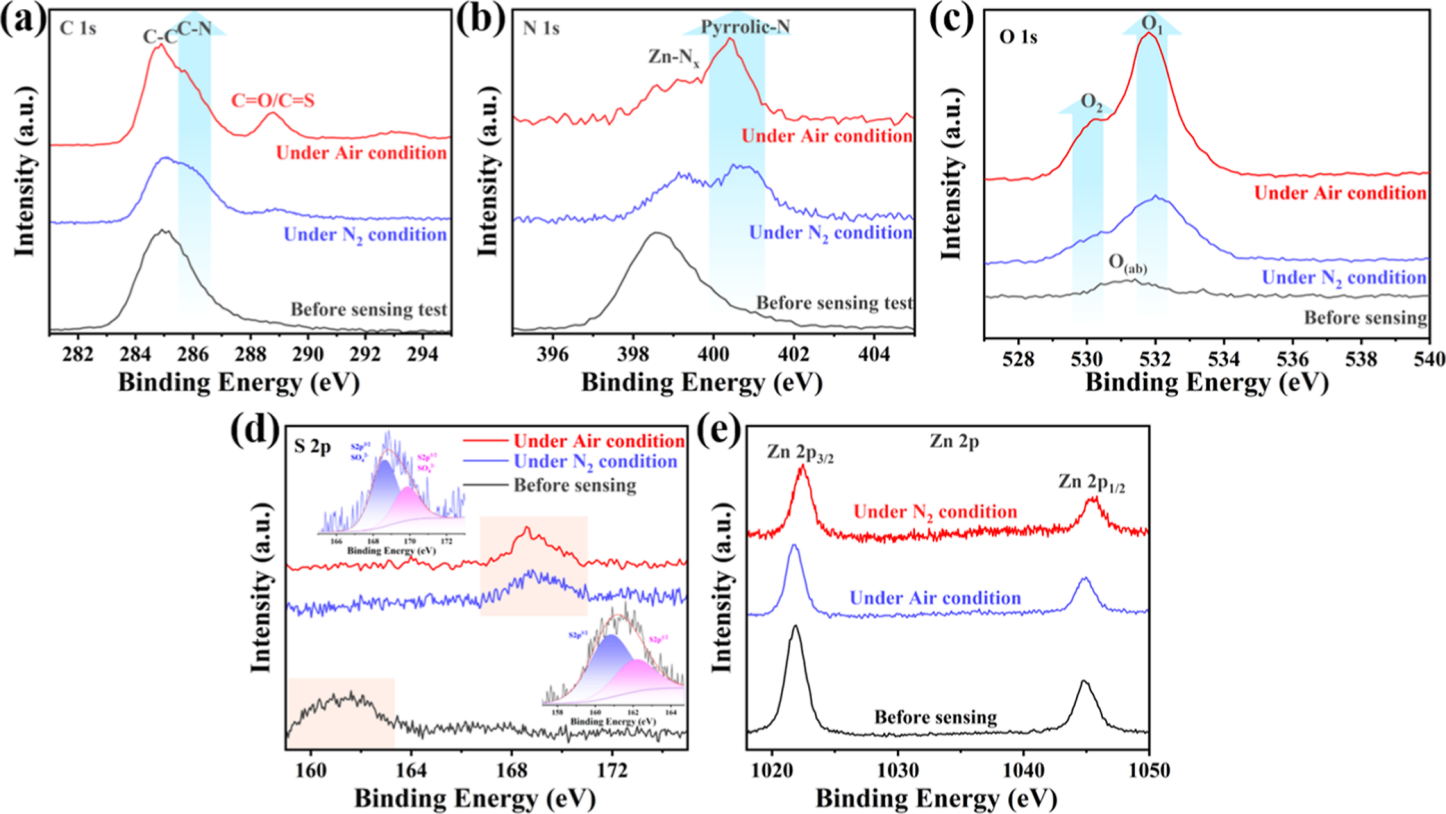
XPS spectra of ZM600 before and after sensing for H_2_S: (a) C 1s spectrum, (b) N 1s spectrum, (c) O 1s spectrum, (d) S 2p spectrum, and (e) Zn 2p spectrum.

Furtherly, to elucidate the role of oxygen in the sensing mechanism, O 1s XPS spectra were analyzed before and after sensing under both N_2_ and air conditions (Figure 6c). The pristine sample exhibited a single peak centered around 531.0 eV, typically attributed to surface-adsorbed oxygen species (e.g., O^2–^, O_2_^–^, O^–^, and hydroxyl groups).^57^ After exposure to H_2_S under N_2_ conditions, O_1_ and O_2_ peaks emerged at approximately 530.2 and 532.0 eV. These signals became significantly more pronounced after sensing under air conditions. The O_1_ peak is assigned to partially oxidized sulfur species, such as sulfite (SO_3_^2–^) intermediates or surface-bound low-valence oxygen generated during the reaction of H_2_S with active oxygen species. The O_2_ peak is consistent with the presence of SO_4_^2–^, indicating that H_2_S underwent partial oxidation even under an oxygen-deficient N_2_ condition.^58^ The intensified signals in the air-exposed sample further confirm the active participation of adsorbed oxygen species (originating from ambient O_2_) in facilitating this oxidation reaction. Furthermore, the S 2p spectra show a clear difference (Figure 6d). Before performing the sensing test and fully exposing the sensor to H_2_S gas, the XPS results revealed two peaks around 161 eV, primarily attributed to S 2p_3/2_ and S 2p_1/2_ in pure H_2_S.^59^ However, after the sensing test, these peaks near 161 eV disappeared, and two new peaks emerged near 169 eV, which were mainly attributed to SO_4_^2–^.^58,60^ The results suggest that Zn–N reacts with H_2_S and adsorbed oxygen on the surface to produce a SO_4_^2–^-based metalate. To investigate the possible changes in the oxidation state of Zn during gas sensing, high-resolution XPS spectra of Zn 2p were collected before and after gas exposure under both air and N_2_ conditions. As shown in the updated Figure 6e, the Zn 2p_3/2_ and Zn 2p_1/2_ peaks in the as-prepared sample are centered at ∼1022.0 eV and ∼1045.0 eV, respectively, which are characteristic of Zn^2+^ species in Zn–N_*x*_ coordination environments.^44^ After exposure to H_2_S under air conditions, the Zn 2p peaks show no significant shift, indicating that the oxidation state of Zn remains stable and that Zn^2+^ retains its coordination environment during sensing. However, under N_2_ conditions, a noticeable shift of approximately +0.44 eV toward a higher binding energy is observed. This shift likely results from changes in the local chemical environment surrounding Zn atoms, possibly due to reduced surface oxygen species and altered electron density under an inert atmosphere. Nonetheless, the persistence of peak positions within the Zn^2+^ binding range confirms that Zn remains predominantly in the +2 oxidation state throughout the sensing process, and no reduction to metallic Zn^0^ is detected.

To clarify whether Zn atoms or clusters are present and contribute to the sensing behavior, high-resolution scanning transmission electron microscope (HRSTEM) imaging was performed (Figure S19). The results indicate that no bright contrast spots were observed, suggesting the absence of Zn atoms or clusters. To gain quantitative insights into the elemental composition and valence state, high-resolution EELS spectra were collected, revealing a weak signal at approximately 383 eV (Figure S20a), corresponding to the Zn M_2,3_-edge and confirming the presence of Zn.^61^ However, no peaks were detected in the Zn L_2,3_-edge region (Figure S20b), suggesting that significant atomic or clustered forms of Zn content are missed.^62^ Additionally, no lattice fringes or contrast patterns of Zn nanoclusters were observed in HRTEM (Figure 2f), further supporting the absence of Zn nanoparticles or clusters. Furthermore, PXRD characterization of ZM600 showed no significant peak broadening or additional features that would be indicative of Zn atoms or clusters (Figure S21), even those below 2 nm in size, which would typically appear near 36.3° and 39.0° (corresponding to Zn(100) and Zn(002)).^63^ XPS analysis further indicates that Zn exists only in a highly coordinated state without any signal corresponding to metallic Zn^0^ (Figure 6e). Collectively, these results provide solid evidence that the enhanced sensing behavior arises from the nitrogen-doped carbon framework and the dispersed Zn–N_*x*_ coordination bonds. Clearly, N sites serve two critical roles: as the primary H_2_S adsorption sites and as crucial bridges connecting Zn and C to form network-like electron transport channels. In this context, N functions like two hands, one holding Zn and the other holding C, simultaneously interacting with H_2_S to enhance the sensor’s performance.

### Gas Sensing Mechanism

Furtherly, to better elucidate the sensing mechanism of ZM600 toward H_2_S, the DFT is employed to investigate the binding energy and charge transfer amount between the gas molecules and Zn–N_*x*_ coordination bonds. The computational details can be found in the Supporting Information. The pure N-doped and Zn–N_*x*_ coordination bonds anchored on N-doped graphitic carbon materials are modeled (Figure 7a), including GR-N, PD-N, PR-N, and their corresponding Zn functionalized bonds (GR-N-Zn, PD-N-Zn, and PR-N-Zn). The binding energy and absolute charge transfer Q between the material and H_2_S molecules are assumed to model the presence of only one type of N on the derived graphene carbon material. Notably, it is almost impossible for the GR-N-Zn to form a proper Zn–N bond, since the graphitic-N is already saturated by the neighboring C atoms. As shown in previous XRD and XPS results, it is found that there is no real presence of Zn isolated atoms, which does not show good catalyst ability. Hence, this case is excluded. The binding configurations between H_2_S and the substrate are searched by the artificial Intelligence for Sustainable Societies (aISS) program.^36^ For each case, 15 configurations are screened out. And the most energy-favorable configuration is selected in the further analysis. The results of computational binding energy and absolute charge transfer of all models are presented in Figure 7b. Both PR-N-Zn and PD-N-Zn yielded larger charge transfer Q and binding energy than the pure-N doped graphitic carbon, which indicates that these two cases could likely generate more significant sensing responses upon exposure to H_2_S. Moreover, the transformation into PD-N will occur upon the adsorption of H_2_S in the case of pure PR-N simulation, where a similar binding energy and charge transfer Q were selected. The above results indicate that the main sensing mechanism is that PR-N and PD-N bonds with Zn mainly adsorb H_2_S and react to generate responses, which validate the experimental results explicitly.

**Figure 7:**
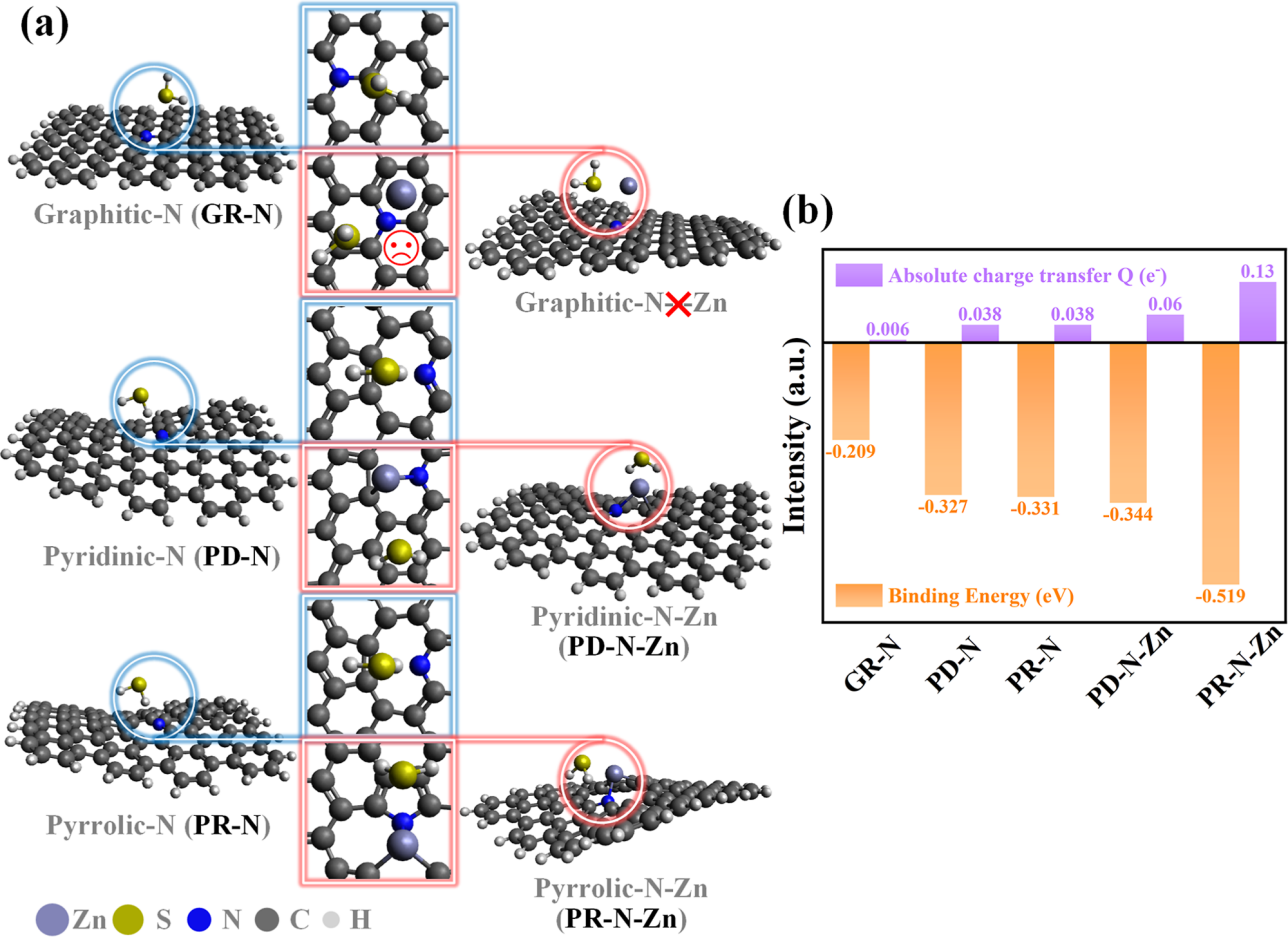
(a) Models of pure N-doped (blue marked) and Zn functionalized N-doped graphitic carbon (light blue marked), including graphitic-N (GR-N), pyridinic-N (PD-N), pyrrolic-N (PR-N), and their corresponding Zn functionalized ones (GR-N-Zn, PD-N-Zn, and PR-N-Zn). (b) Binding energy and absolute charge transfer Q of ZM600 toward H_2_S.

Consequently, the computational results concretely demonstrate that the experimental N, which can be clearly identified as PR-N and PD-N, are major contributors in sensing application. The synergistic reaction mechanisms for H_2_S gas sensing based on the experiment and computational results are proposed and depicted in Figure 8:

(1) The derived N-doped graphitic carbon materials exhibit fitted electrical conductivity and contain a large number of active sites on their surfaces. This is attributed to the nitrogen doping into the graphitic carbon structure in two ways: one is working as donors, introducing lone electrons into the lattice carbon, and the other is working as acceptors, introducing positively charged holes into the originally stable lattice carbon.^64,65^

(2) PR-N-Zn and PD-N-Zn on N-doped graphitic carbon materials act as the primary gas adsorption sites, where Zn atoms (with oxidation states ranging from 0 to +2) typically provide an empty orbital, considered as positively charged holes, while the surrounding N atoms act as donors providing negatively charged electrons. In H_2_S molecules, H^+^ is oxidative, while S has the lowest oxidation state and is not oxidative, which indicates that H^+^ readily acquires free electrons from the material surface or electrons provided by N to turn into H_2_, S^2–^ with oxygen groups adsorbed on the surface to form SO_4_^2–^.
(3) The PR-N-C and PD-N-C functionalities in N-doped graphitic carbon materials interact with H_2_S, releasing H_2_ and leading to the formation of new carbon-based surface groups. A characteristic signal at ∼289 eV emerges after H_2_S exposure, which may arise from C=O or C=S bonding environments. Despite the challenge in unambiguously distinguishing between these species, their presence reflects notable chemical modifications at the carbon sites. These transformations result in altered electronic structures and local charge redistribution, thereby enhancing carrier concentration and contributing to the observed sensing response.
(4) Compared to pure PR-N-Zn and PD-N-Zn coordination bonds and N-doped graphitic carbon structures, the PR-N-Zn and PD-N-Zn coordination bonds anchored on N-doped graphitic carbon exhibit stronger abilities to reduce oxygen molecules to superoxide species due to their multichannel electron transport advantages. Additionally, nitrogen functionalized as both donors and acceptors effectively bridges the gap between Zn and C, opening up new electron transport channels.
(5) Oxygen molecules adsorbed on the sensor surface capture free electrons from the material surface, generating superoxide species. When H_2_S gas molecules are adsorbed onto the material surface, they react with superoxide species, releasing electrons instead of other species. This is because the resistance of the sensor increases with the introduction of H_2_S gas, indicating a decrease in the thickness of the electron-depleted layer with injected electrons.

**Figure 8:**
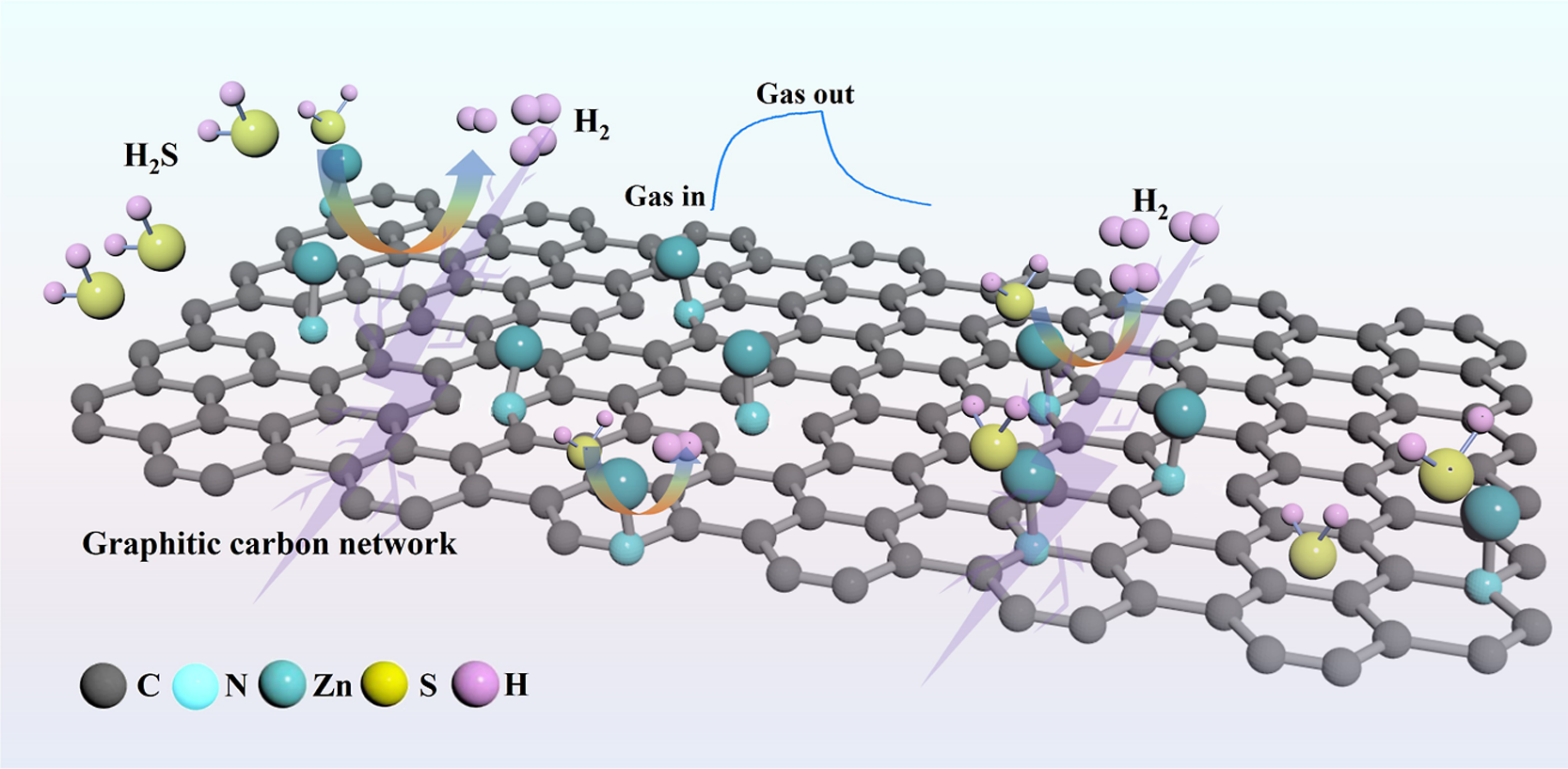
Proposed gas sensing mechanism of PD-N-Zn and PR-N-Zn functionalized N-doped graphitic carbon toward H_2_S.

## Conclusions

In this work, PD-N-Zn and PR-N-Zn functionalized N-doped graphitic carbon was first studied and applied for improving gas sensitivity and selectivity toward trance H_2_S detection, demonstrating excellent sensing performance toward H_2_S gas with an LOD of 56.9 ppb, faster response and recovery time (18 s and 29 s), and high selectivity with a 20 folds response difference than other interfering gases. The expected stability with stable multiple consecutive responses and a strong response toward 1 ppm of H_2_S after 4 months were reached. The results suggest that PR-N and PD-N sites in N-doped graphitic carbon play dual roles in the H_2_S sensing mechanism. On the one side, N–C bonds interact with H_2_S, leading to H_2_ release and the formation of new carbon-based bonding environments—evidenced by the emergence of a ∼289 eV C 1s peak, which may originate from C=O or C=S species. The subsequent structural rearrangement enables the formation of additional C–N bonds at unsaturated carbon sites, introducing new adsorption centers. On the other side, PR/PD-N-Zn coordination motifs participate in redox interactions with H_2_S and surface-adsorbed oxygen, facilitating the formation of SO_4_^2–^-based metalate species. Notably, the DFT calculation was employed to confirm both PR-N and PD-N bonding with zinc, yielding the largest charge transfer and binding energy among simulated factors, which attribute to generate significant sensing performance for H_2_S. The formation of transport paths among Zn, N, and C and the generation of synergistic effects among Zn and surrounding N and between N and C both absolutely support sensing application. Consequently, this work will provide a novel strategy for the advancement of gas sensing applications.

## Supplementary Material



## Notes

Additional material characterization including SEM, XRD, TEM, EELS, BET, and Raman spectra, as well as extended gas sensing performance data (PDF)
