# Supplementary material for: Highly Sensitive and Selective Zinc-Based Metal–Organic Framework Derivatives Gas Sensors for Trace H2S Detection
Source: ACS Sens. 2025 Oct 2;10(10):7584–98. doi: 10.1021/acssensors.5c01743 (PMC12560126; doi:10.1021/acssensors.5c01743)
Supplement: Supplementary file 1 [file se5c01743_si_001.pdf]

Supporting Information

Highly Sensitive and Selective Zinc-Based Metal-Organic Framework Derivatives Gas Sensors for Trace H_2S Detection

Wei Wang, [1] Li Chen, [1] Leif Riemenschneider, [1] Chen-Chen Wang, [1,2,3] Luis-Antonio Panes-Ruiz, [1] Martin Hantusch, [4] Yun-Xu Chen, [5] Jian-Jun Zhang, [6] Shivam Singh, [4,7] Yana Vaynzof, [4,7] Markus Löffler, [8] Arezoo Dianat, [1] Naisa Chandrasekhar , [6] Shi-Rong Huang, *[1] and Gianaurelio Cuniberti *[1,9]

[1] Institute for Materials Science and Max Bergmann Center for Biomaterials, TUD Dresden University of Technology, 01062, Dresden, Germany

[2] University of Strasbourg, Institute Charles Sadron, CNRS, UPR22, 23 Rue du Loess, 67034, Strasbourg Cedex 2, France

[3] Faculty of Chemistry and Food Chemistry, Technische Universität Dresden, 01062, Dresden, Germany

[4] Leibniz-Institute for Solid State and Materials Research (IFW), 01062, Dresden, Germany

[5] Max Planck Institute for Microstructure Physics, 06120, Halle (Saale), Germany

[6] Center for Advancing Electronics Dresden (cfaed) and Faculty of Chemistry and Food Chemistry, TUD Dresden University of Technology, 01062, Dresden, Germany

[7] Chair for Emerging Electronic Technologies, TUD Dresden University of Technology, 01187, Dresden, Germany

[8] Dresden Center for Nanoanalysis (DCN), Center for Advancing Electronics Dresden (cfaed), TUD Dresden University of Technology, 01069, Dresden, Germany

[9] Dresden Center for Computational Materials Science (DCMS), TUD Dresden University of Technology, 01062, Dresden, Germany

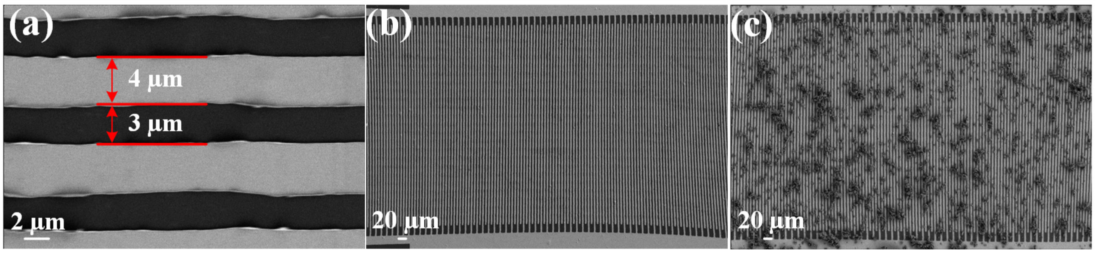

Figure S1. (a-c) Gold electrode sensor device: before (a-b) and after (c) drop casting of as-prepared samples.

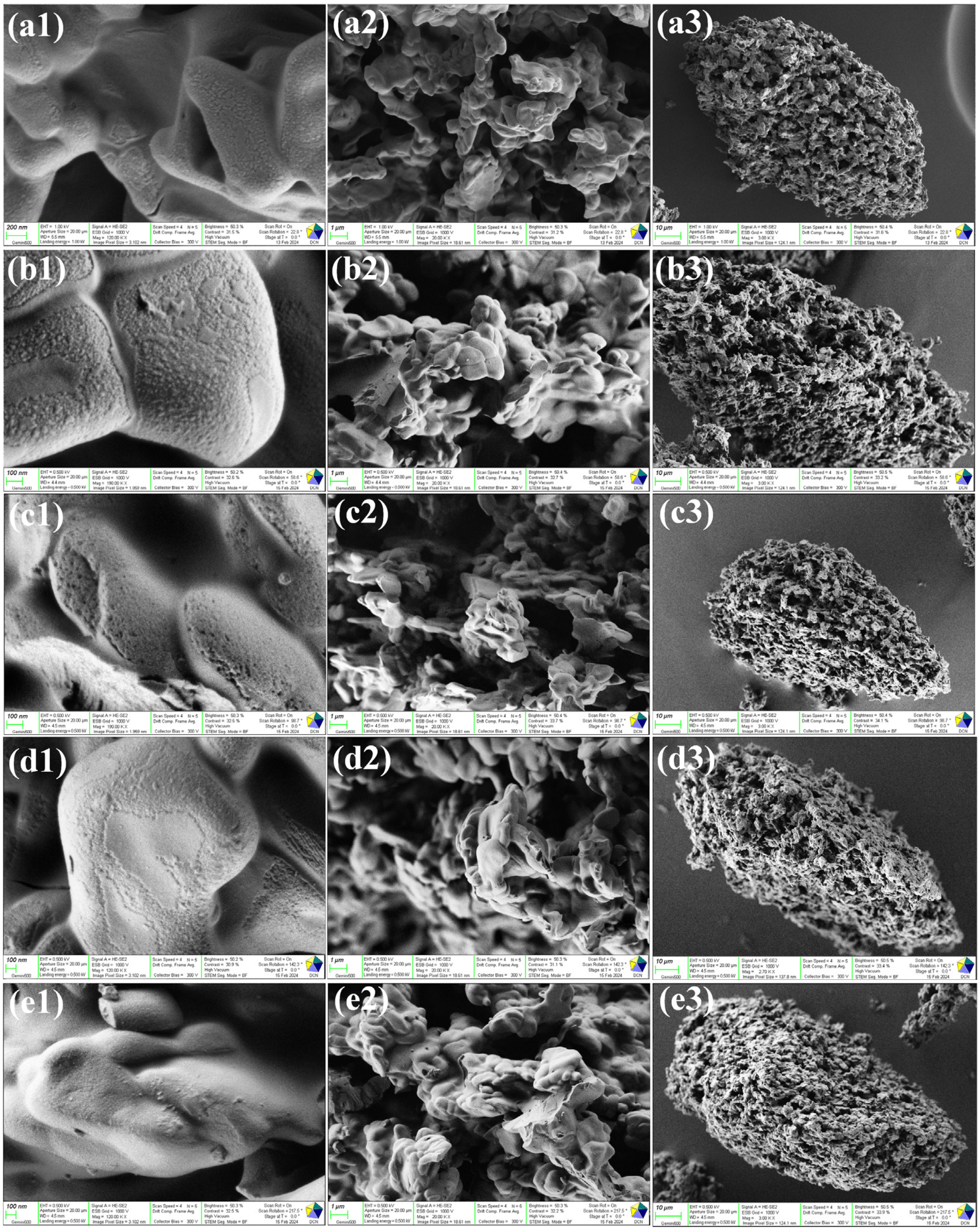

Figure S2. (a-e) SEM images: ZM400 (a1-a3), ZM500 (b1-b3), ZM600 (c1-c3), ZM700 (d1-d3) and ZM800 (e1-e3).

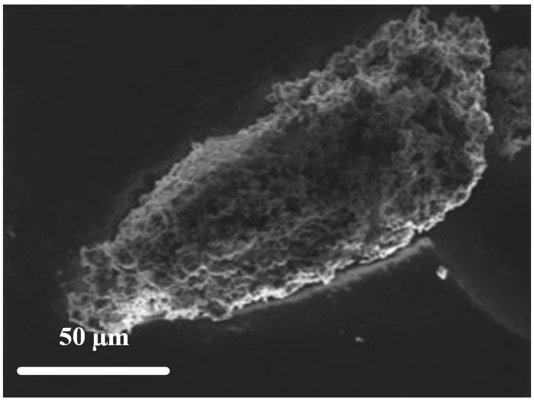

Figure S3. SEM image for EDX mapping of ZM600.

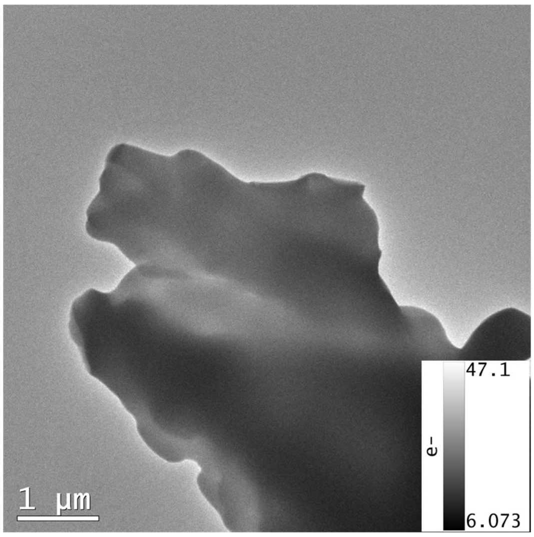

Figure S4. TEM image of ZM600.

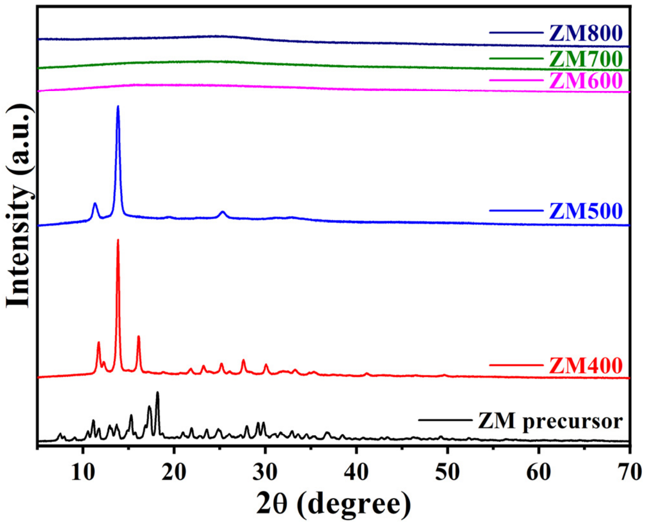

Figure S5. XRD patterns of ZM precursor, ZM400, ZM500, ZM600, ZM700 and ZM800.

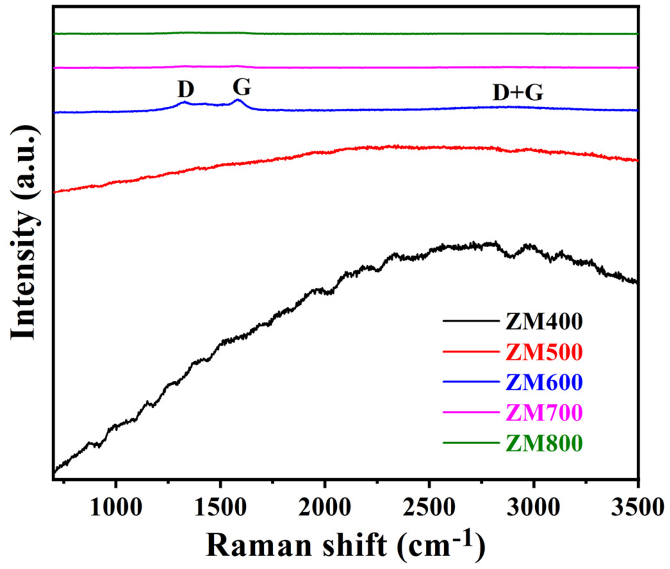

Figure S6. Raman spectra of ZM400, ZM500, ZM600, ZM700 and ZM800.

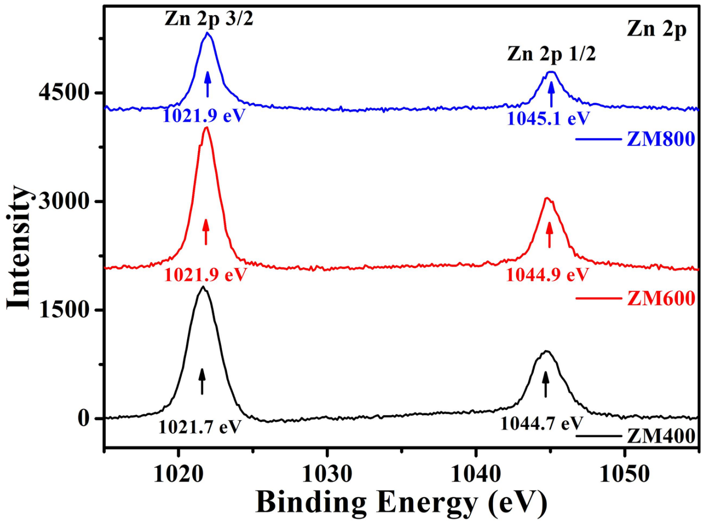

Figure S7. XPS Zn 2p spectra of ZM400, ZM600, and ZM800.

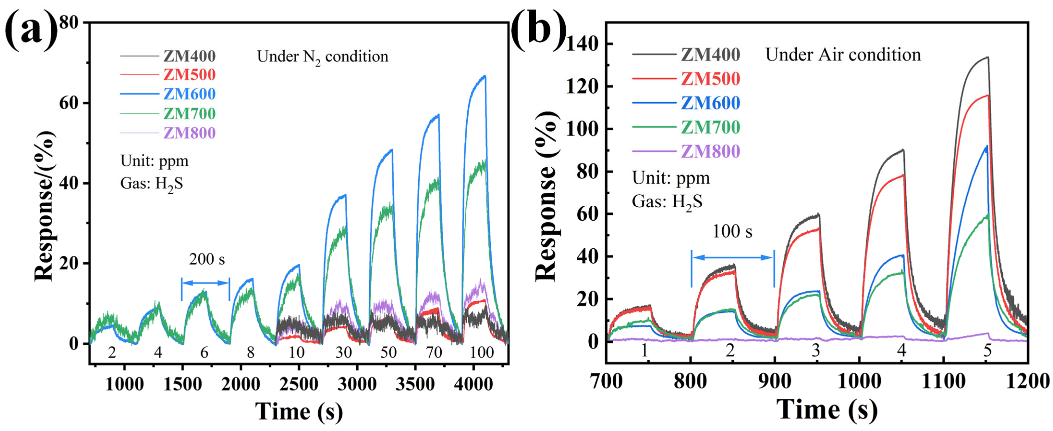

Figure S8. (a-b) Sensing performance at 200 °C operating temperature: Response and recovery curves towards H_2S with different concentrations under N_2 condition (a) and Air condition (b) for

all prepared samples.

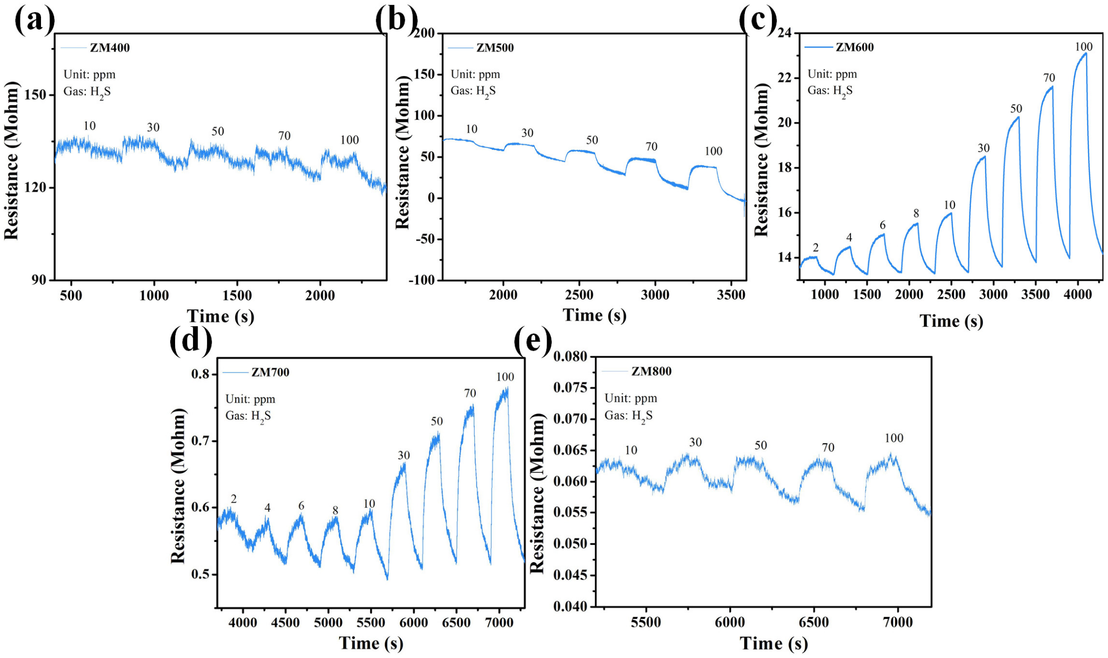

Figure S9. (a-e) Resistance varying curves towards H_2S with different concentrations under N_2 condition: ZM400 (a), ZM500 (b), ZM600 (c), ZM700 (d), ZM800 (e).

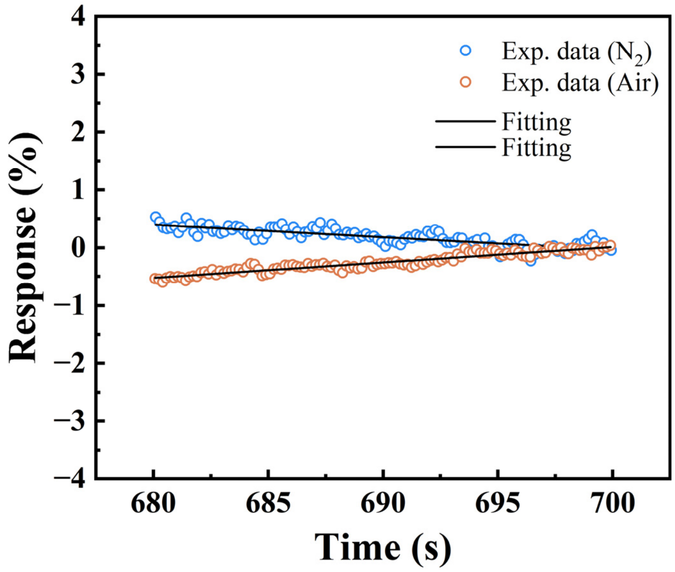

Figure S10. RMS noise levels of response as a function of time under N_2 and air conditions for ZM600.

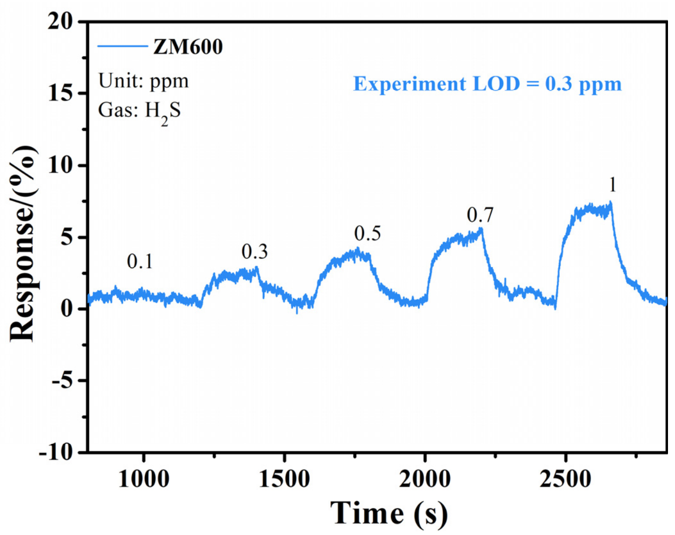

Figure S11. Response and recovery curves towards H_2S with lower concentrations under N_2 condition for ZM600.

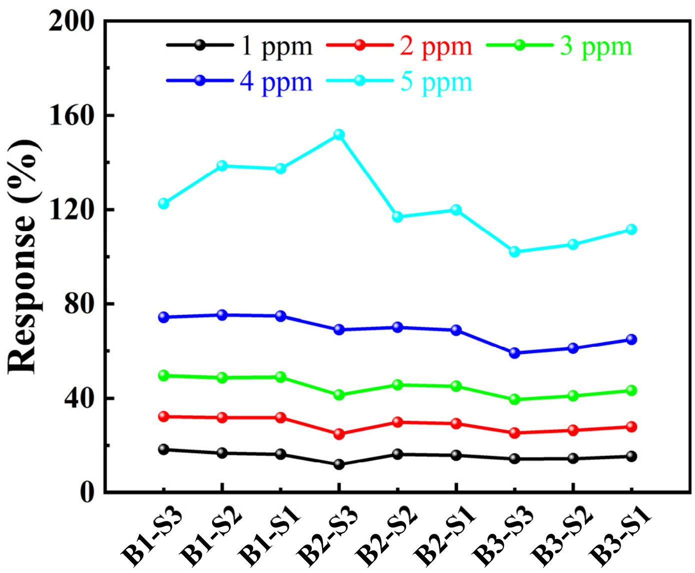

Figure S12. Comparison of sensing performance of three sensors from each of three independent batches of ZM600 material towards different concentration of H_2S.

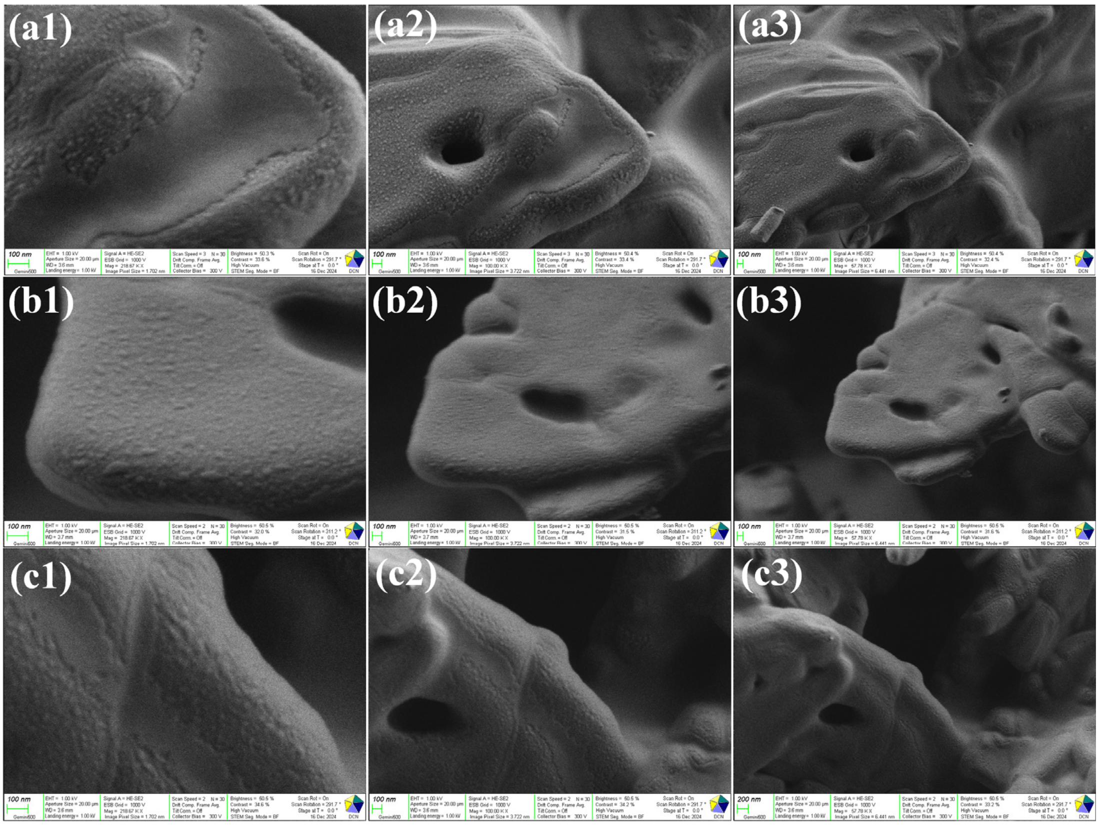

Figure S13. (a-c) SEM images: batch 1 of ZM600 (a1-a3), batch 2 of ZM600 (b1-b3), batch 3 of ZM600 (c1-c3).

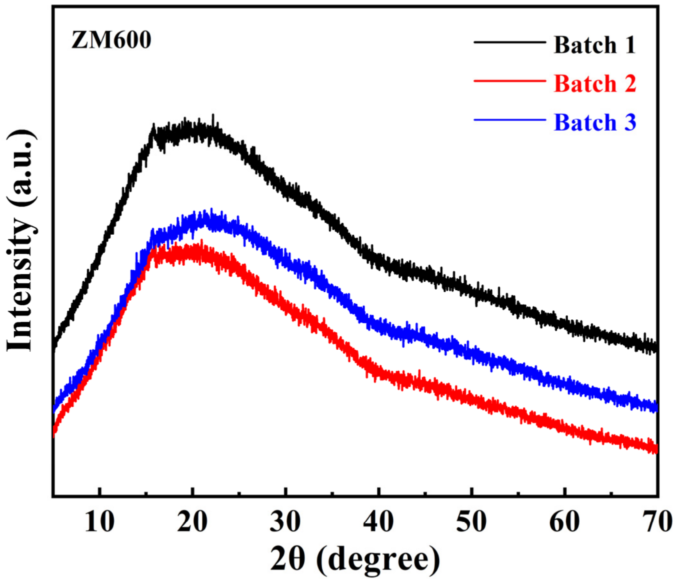

Figure S14. XRD pattern of three batches of ZM600.

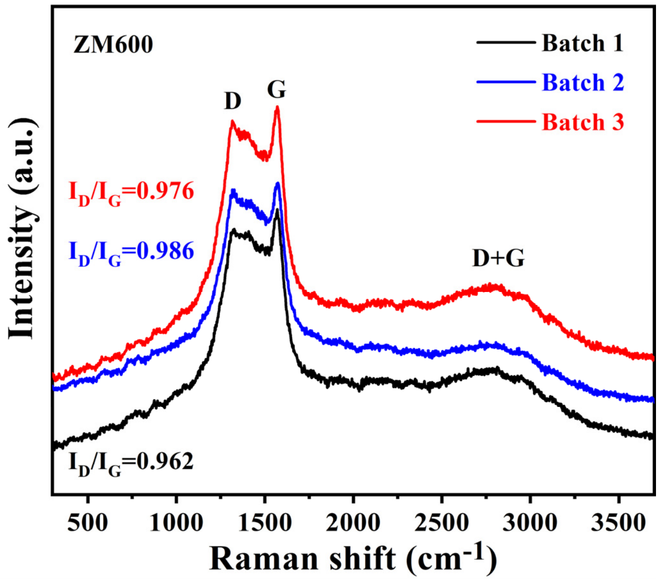

Figure S15. Raman pattern of three batches of ZM600.

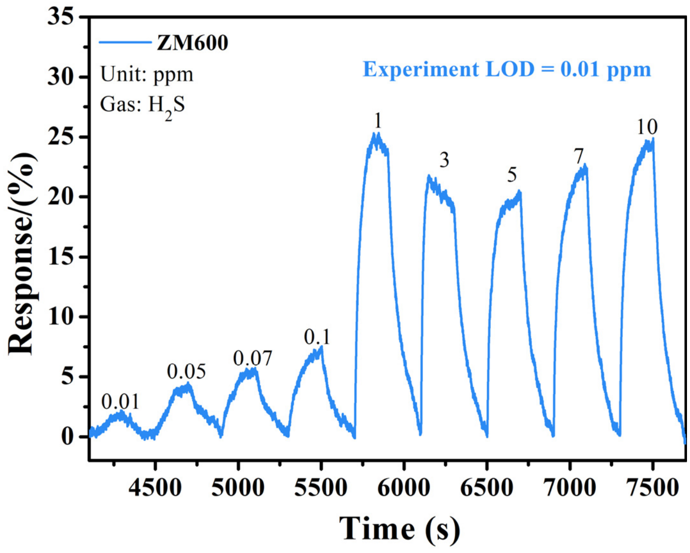

Figure S16. Response and recovery curve towards H_2S with different concentrations under N_2 condition for ZM600 after 120 days.

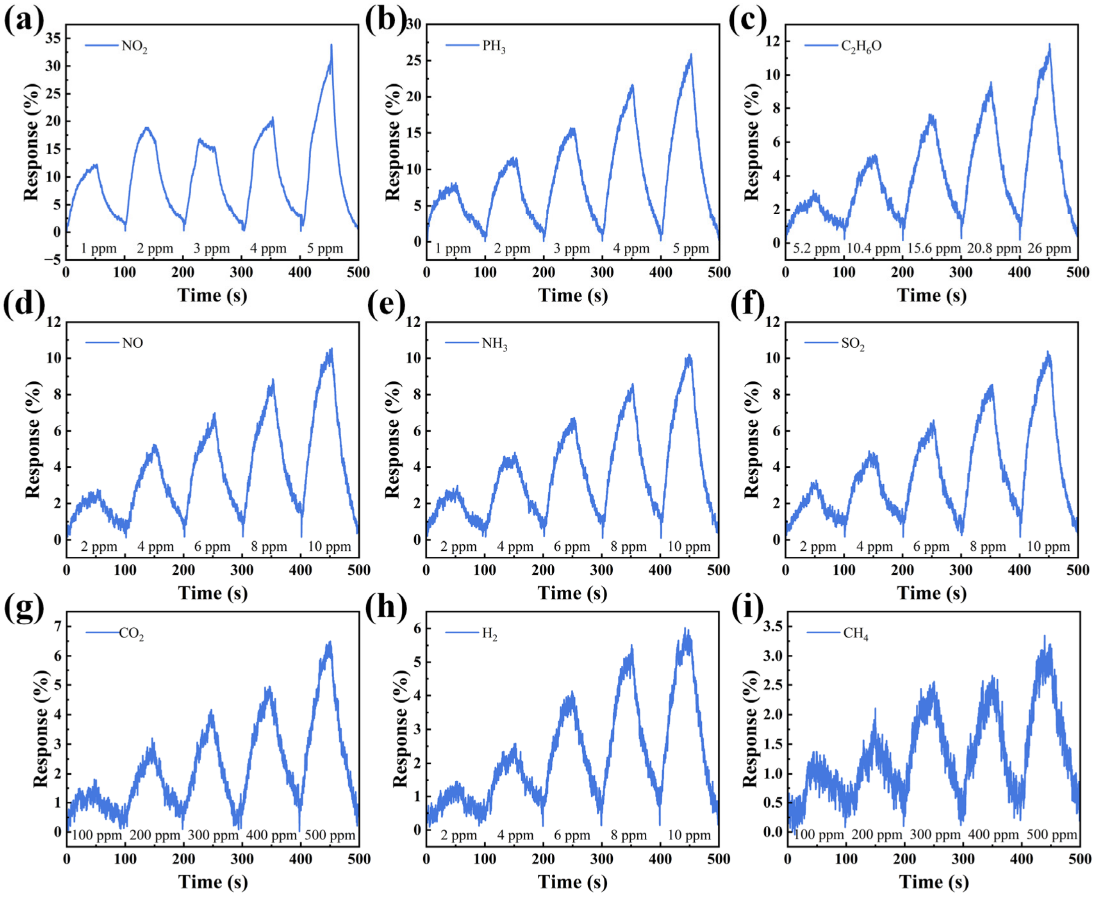

Figure S17. Response and recovery curves of ZM600 towards different interfering gases at different concentration: (a) NO_2, (b) PH_3, (c) C_2H_6O, (d) NO, (e) NH_3, (f) SO_2, (g) CO_2, (h) H_2, and (i) CH_4.

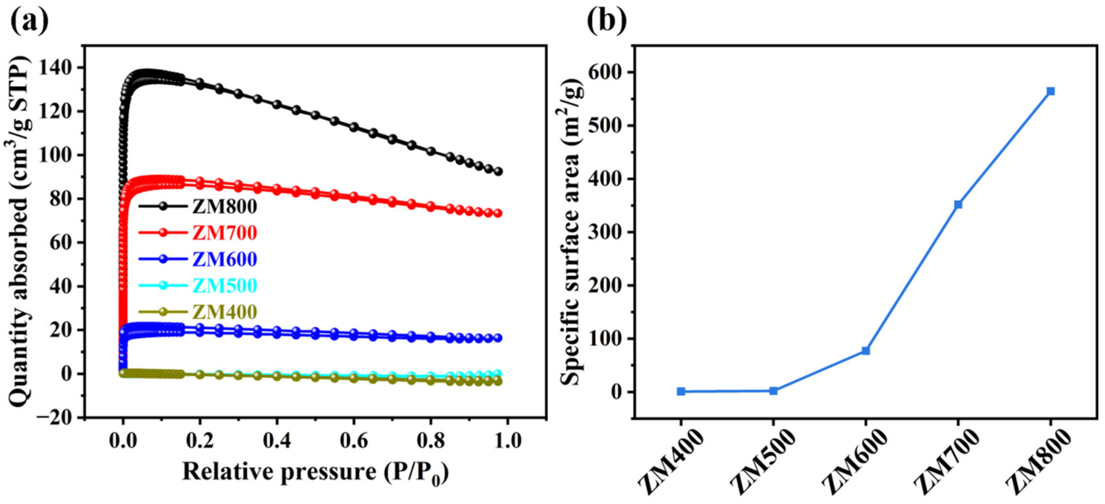

Figure S18. (a) N_2 adsorption–desorption isotherms. (b) BET specific surface area for all samples.

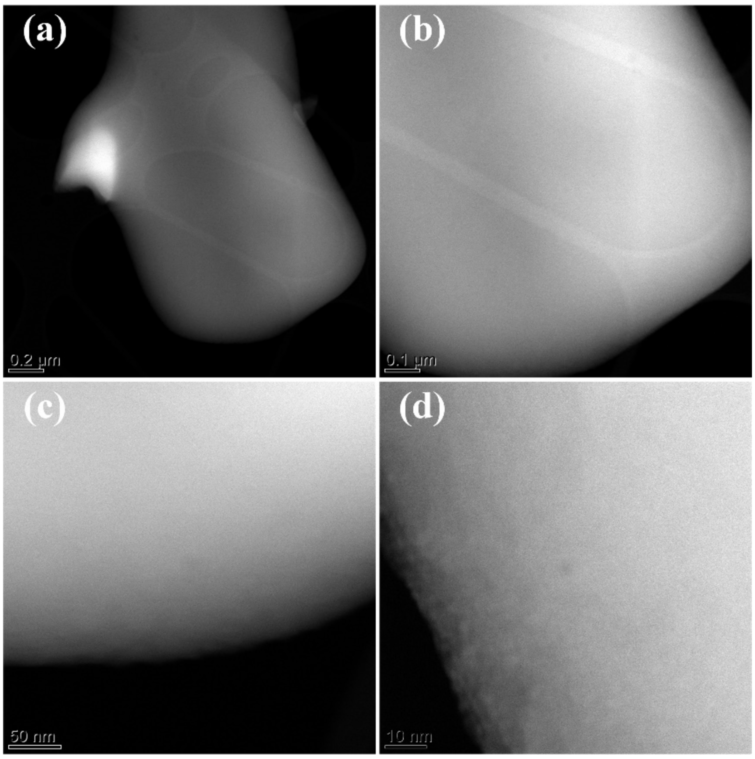

Figure S19. HRSTEM images of ZM600 sample (scale: a 0.2 μm; b 0.1 μm; c 50 nm; d 10 nm).

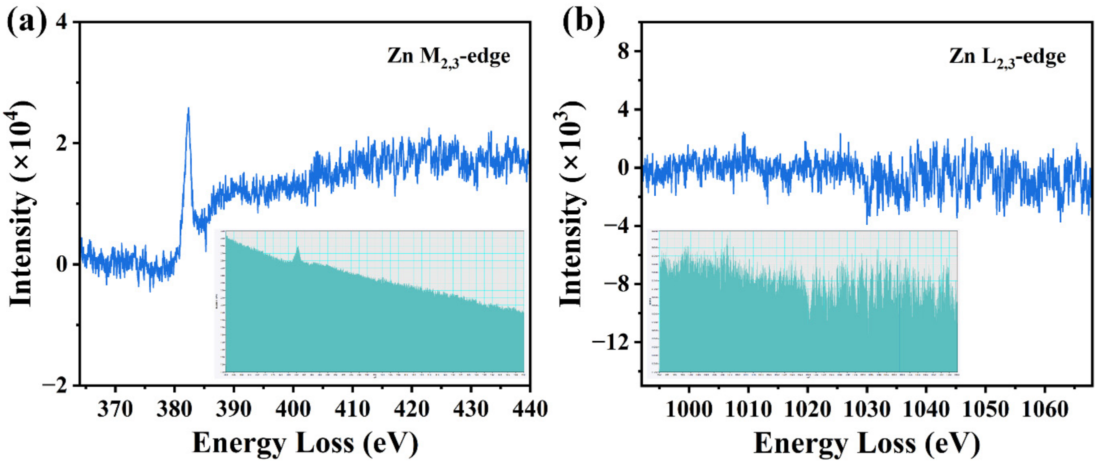

Figure S20. The high-resolution EELS profiles of the ZM600 sample.

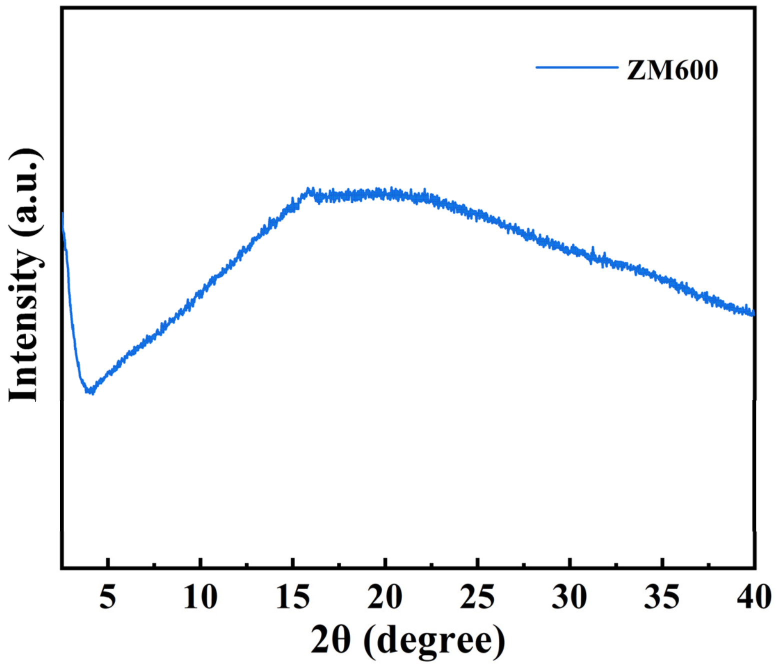

Figure S21. PXRD pattern of ZM600 sample (2θ: from 2.5 to 40 degree).
